# Clinical Effectiveness of Traditional Polyherbal Formulations for Wound Healing: A Systematic Review and Meta‐Analysis

**DOI:** 10.1155/tswj/1551004

**Published:** 2026-05-11

**Authors:** Samuel Abiodun Kehinde, Nurulhusna Awaeloh, Pinanong Na-Phatthalung, Siriwan Kantisin, Sasitorn Chusri

**Affiliations:** ^1^ Biomedical Technology Research Group for Vulnerable Populations and the School of Health Science, Mae Fah Luang University, Muang Chiang Rai, Thailand, mfu.ac.th; ^2^ Biochemical/EnTox Lab, Department of Environmental Health Science, Faculty of Basic Medical Sciences, Ajayi Crowther University, Oyo, Oyo State, Nigeria, acu.edu.ng; ^3^ Thai Faculty of Allied Health Science, Nakhon Ratchasima College, Nakhon Ratchasima, Thailand; ^4^ Division of Hematology and Oncology, Icahn School of Medicine at Mount Sinai, New York, New York, USA, mountsinai.org; ^5^ Occupational Health and Safety Program, School of Health Science, Mae Fah Luang University, Chiang Rai, Thailand, mfu.ac.th; ^6^ Area-Based Research and Innovation in Cross-Border Health Care Group, Mae Fah Luang University, Chiang Rai, Thailand, mfu.ac.th

**Keywords:** chronic wounds, diabetic ulcers, herbal medicine, inflammation, medicinal herbs, polyherbal, tissue regeneration, wound healing, wound management

## Abstract

Chronic wounds, especially in diabetic patients, are difficult to manage because of delayed healing and high infection risk. Traditional polyherbal formulations, which are composed of multiple medicinal plants with synergistic effects, are widely used in wound care, but their clinical effectiveness has not been comprehensively synthesized. This study aimed to perform a systematic review and meta‐analysis of randomized controlled trials (RCTs) assessing the effectiveness of traditional polyherbal formulations for wound healing. Databases were searched, and RCTs involving adults with wounds, where the intervention was traditional polyherbal formulation rather than a placebo, standard care, or other active treatments, were included. The meta‐analysis was conducted via a random‐effects model, and the certainty of evidence was evaluated via GRADE. Eight RCTs were included. The pooled mean difference for healing time favored polyherbal formulations (−3.28 days; 95*%*CI = −8.56 to 2.01) but was not statistically significant. Similarly, HbA1c reduction (−5.97%; 95*%*CI = −30.86 to 18.93) was not significantly different, with high heterogeneity (*I* ^2^ = 98*%*), and no publication bias was detected. Although several individual herbs within these formulations possess tissue‐regenerative, angiogenic and anti‐inflammatory properties, the pooled results indicate only a modest, nonsignificant trend toward faster healing. Variations in formulation composition, treatment duration, and methodological quality limit the certainty of evidence, which ranges from high (age) to very low (HbA1c) on the GRADE assessment. Overall, polyherbal formulations show therapeutic promise as adjuncts to standard wound, whereas larger, well‐designed trials using standardized formulations and clinically relevant endpoints are needed to establish their efficacy and optimize their clinical application.

## 1. Background

The wound‐healing process is a multistage, dynamic physiological process, which is necessary to restore skin integrity after injury. It comprises four overlapping phases that are interdependent and include hemostasis, inflammation, proliferation, and remodeling [1]. The productivity and balance of these phases are critical toward fast and effective tissue repair. Although acute wounds are basically expected to heal in a predictable fashion, chronic wounds including diabetic foot ulcer, venous leg ulcer, and pressure sore often exhibit slow healing response, thus, leading to high morbidity of the patient, risks of infection, risks of amputation of limbs, and high costs of care [2, 3]. Chronic wounds have been observed to afflict over 40 million people in the world with diabetic foot ulcers alone causing a rate of about 15% of diabetic patients during their lifetime [4]. The financial consequences are significant. Annually, the expenditure on chronic wound treatment in the United States is estimated to be more than $25 billion with similar economic influences being experienced in the United Kingdom and other advanced countries [5]. In addition to financial impacts, patients experience chronic pain, loss of ability, mental agony, and deteriorate quality of life. In this regard, there is an urgent need to have therapeutic interventions that are effective and accessible, but at a low cost.

In many environments, despite the existence of modern wound‐care techniques such as surgical debridement, advanced dressings, skin grafting, negative‐pressure wound therapy, as well as growth‐factor based products, clinical outcomes are not as optimal as they could be. Among the limitations of these modalities are high cost, inaccessibility in resource‐limited settings, negative responses, and low efficacy in complicated wound settings [6, 7]. On the one hand, biologic agents such as recombinant human platelet‐derived growth factor (rhPDGF) have demonstrated potential in improving wound healing, but their use in clinical practice is constrained by prohibitive cost, cold‐chain needs, and regulatory barriers [8]. Antibiotics, which are routinely used to prevent or treat wound infections, contribute to the rising rate of resistance and dysregulation of the microbiome [9]. More so, modern wound dressings despite being technologically superior are not always effective in all wound types and in most cases are unable to treat the multifactorial pathophysiology of chronic wounds. These issues have highlighted the need to consider other alternative, complementary, and integrative methods which are cost‐effective, culturally acceptable, and can have the ability to tune multiple pathways of healing at the same time.

Polyherbal preparations have been used in traditional medicine, such as Ayurveda, traditional Chinese medicine (TCM), Siddha, and Unani to heal wounds, and many such preparations are polypharmacy by combining several botanicals to produce synergistic therapeutic effects. Polyherbalism is based on the fact that a mixture of herbs can be used to address multiple biological processes during wound repair including, but not limited to, the regulation of inflammation, angiogenesis, proliferation of fibroblasts and collagen deposition, as well as antimicrobial effect [10, 11]. Multipotent mechanisms of action, low systemic toxicity, and cultural acceptability of polyherbal preparations in the areas where traditional medicine is a primary or adjunct form have led to their popularity. There is also accumulating scientific information that their pharmacological importance lies in their ability to regulate wound healing at both molecular and cellular levels, such as cytokines, oxidative stress, angiogenic factors, and extracellular matrix remodeling [11].

Although polyherbal formulations have been shown to be effective to treat wounds, numerous preclinical trials and anecdotal clinical reports have been reported; high‐quality, evidence‐based clinical trials are relatively long to come by. It has been shown in the recent clinical practice that polyherbal preparations may be used to improve wound healing in humans. A small‐scale test of a Thai polyherbal extract (T‐YaSP) has indicated much better percentages of full recovery of diabetic wounds compared to the conventional therapy [12]. Similarly, Ayurvedic preparations like *Jatyadi Taila* and *Panchavalkala* sprays have also been shown to have the same or better effect on chronic ulcers using multicenter clinical trials [13, 14]. Topical use of Dragon′s blood polyherbal cream in a randomized, double‐blind trial hastened wound contraction and epithelialization compared with placebo [15, 16]. Other pilot and open studies have also shown significant reductions in wound size, pain, and time to re‐epithelialization [17]. These results taken together offer preliminary clinical evidence on the therapeutic role of polyherbal preparations in wound management, but the differences in the quality and methodology of the studies indicate the importance of a complex synthesis of the topic through systematic review and meta‐analysis. Most of the reviews that have been carried out have looked at individual herbs or individual traditional systems less than polyherbal combinations, thus, restricting the scope of interpretation [18–20]. Additionally, methodological limitations in previous systematic reviews such as small sample sizes, use of heterogeneous outcome measures, absence of blinding, and lack of risk‐of‐bias assessment have been common. A lot of literature available are not consistent in their analysis of standardized endpoints like time to close wound, wound reduction, wound infection, and histopathologic parameters.

What makes the situation more complicated is that quantitative meta‐analytical frameworks of data pooling and obtaining statistically significant estimates about the efficacy of the treatment are hardly integrated. Without stringent synthesis, clinicians and political authorities would struggle to make informed choices as far as the integration of polyherbal therapies in conventional wound‐care guidelines is concerned. In turn, a systematic review and meta‐analysis that would cover the effectiveness of traditional polyherbal preparations in the clinical setting are timely and required. The current systematic review and meta‐analysis attempt to fill the knowledge gap that exists through a critical appraisal and synthesis of the available randomized controlled trials (RCTs), which have studied the clinical efficacy of traditional polyherbal formulations in wound healing. The study aims to inform clinicians, researchers, and policymakers who may be interested in integrative wound management by supplying a scientifically sound evidence base. The results are supposed to guide the future research directions, facilitate the rational use of traditional medicine, and possibly lead to creating the affordable, culturally acceptable, and effective wound‐care solution on the global level. Critically, the intent of this review is not to position polyherbal formulations as replacements for evidence‐based standard wound care (SWC), which includes wound debridement, appropriate dressings, infection management, and offloading in diabetic patients, but rather to evaluate their potential as adjunctive interventions that may enhance or accelerate healing outcomes when integrated with current best practice. Understanding the incremental benefit of these formulations, if any, above and beyond standard care is the central clinical question guiding this synthesis.

## 2. Methods

### 2.1. Study Design and Protocol Registration

**Figure 1:**
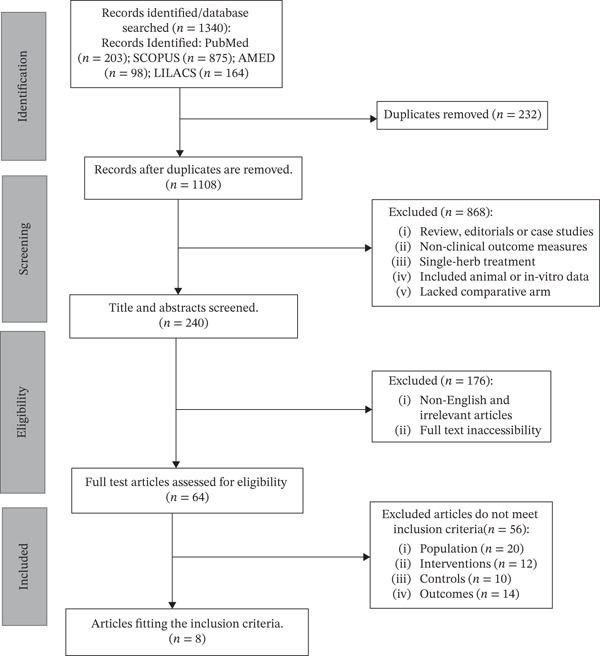
PRISMA flow diagram of the systematic review process.

The systematic review was developed according to the PRISMA guidelines of systematic reviews and meta‐analyses [21]. Figure 1 depicts the PRISMA flow diagram of the systematic review process. The protocol of the systematic review was preregistered in PROSPERO (CRD420251125531). An analysis of the derived data in terms of population characteristics and clinical efficacy parameters of wound healing (age, duration of diabetes, duration of wounding, baseline wound size, wound‐healing time, and level of HbA1c) was conducted using the meta‐analysis online (http://metaanalysisonline.com, https://http://metaanalysisonline.com; accessed August 12, 2025 [22]).

### 2.2. Information Sources and Search Strategy

Without any time restrictions, databases such as PubMed (http://http://www.pubmed.gov), Scopus (http://http://www.scopus.com), AMED (https://http://www.ebsco.com/productsresearch- databases/amed), and LILACS (https://http://www.lilacs.bvsalud.org) were searched. Findings that were published through these databases until August 12, 2025, were searched. Various descriptors were used, which included grouped Boolean operators and terms like wound healing, chronic wounds, ulcer healing, polyherbal formulation, herbal combination, traditional medicine, Ayurvedic medicine, TCM, Unani, clinical trial, and treatment outcome, to locate relevant studies. The descriptors were combined using Boolean operators “AND” and “OR.” Each database had a search strategy that was custom‐made and sensitive/specific. The complete database‐specific search strings for all four databases, including MeSH terms, keywords, and Boolean operators, are provided in their entirety in Table S1 to ensure full reproducibility of the search strategy. No additional hand‐searching of journal issues or conference proceedings was performed, and searches of gray literature databases (e.g., OpenGray, ProQuest Dissertations, and Theses) were not undertaken. We therefore limited study identification to the records retrieved from the electronic databases above.

### 2.3. Eligibility Criteria

Studies on the basis of the following predefined eligibility criteria, which were structured according to the PICOS framework, were included (Table 1).

**Table 1:** Eligibility criteria for the selected studies.

Component	Description
Population (P)	Adults (≥ 18 years) with acute or chronic wounds of any etiology, including diabetic foot ulcers, venous leg ulcers, pressure ulcers, traumatic wounds, or surgical wounds.
Intervention (I)	Administration of a traditional polyherbal formulation, either oral, topical, or combined, derived from recognized systems such as Ayurveda, traditional Chinese medicine (TCM), Siddha, Unani, or other ethnomedicinal practices. The formulation must contain two or more herbal ingredients.
Comparator (C)	Standard wound care, placebo, monotherapy herbal treatment, or other conventional or alternative wound‐healing interventions.
Outcomes (O)	Primary outcomes: complete wound‐healing rate, percentage reduction in wound size or area, and time to complete healing. Secondary outcomes: infection rate, recurrence rate, pain reduction, histopathological improvement, and adverse events.
Study design (S)	Randomized controlled trials (RCTs) were included.

### 2.4. Inclusion Criteria

Eligible studies included RCTs involving adults (≥ 18 years) with acute or chronic wounds of any cause, where a traditional polyherbal formulation (containing at least two herbal ingredients and administered orally, topically, or both) was compared with standard care, placebo, monotherapy herbal treatment, or other interventions, and reported outcomes such as wound‐healing rate, reduction in wound size, time to healing, infection or recurrence rates, pain reduction, histopathological changes, or adverse events.

### 2.5. Exclusion Criteria

1. Investigated single‐herb treatments;
2. Included animal or in vitro data only;
3. Lacked a comparative arm;
4. Were reviews and editorials;
5. Used nonclinical outcome measures exclusively.

### 2.6. Prognostic Criteria

To enhance interpretability and contextualize heterogeneity across studies, the most significant prognostic determinants that are known to affect wound‐healing outcomes were determined and evaluated regardless of the intervention applied. These factors were used to evaluate comparability of the baseline between the groups of the study and to frame differences in the reported outcomes. The type and etiology of the wound were considered to be the key determinants of the healing response. Some of the types of included wounds included diabetic foot ulcers, venous leg ulcers, pressure injuries, and acute traumatic wounds. Delayed healing is linked with chronic wounds especially diabetic patients due to prolonged inflammation, neuropathy and ischemia. The size and depth of the baseline wounds were also investigated because larger and deeper wounds, where the wound size exceeds 5 cm^2^ in size, are associated with extended healing periods.

The time interval taken before an intervention was another critical prognostic determinant. Injuries that last longer than 30 days were considered more recalcitrant, and they showed signs of very complicated healing. In diabetic populations, glycemic control measures, that is, the HbA1c levels where mentioned, were examined since both are directly related to wound‐healing processes and predisposition to infection. Biofilm or wound infection, which is often underreported, was taken as a negative prognostic factor. This was of specific relevance in literature assessing polyherbal preparations with proven antimicrobial effects, such as *Curcuma longa* and *Azadirachta indica*. Patient‐specific factors, such as age, smoking, dietary status, and comorbidity including peripheral vascular disease, were also noted because of their possible effect on the healing process.

In instances where it was necessary, the severity of ulcers was befriended by applying the Wagner or NPUAP classification which are the standardized stages of ulcers. Besides this, previous or concomitant wound‐care interventions, such as debridement, offloading plans, and antibiotic therapy, were recorded to be the potential confounding variables. Taken together, the analysis of these prognostic factors allowed having a more subtle comprehension of clinical outcomes and facilitated a better understanding of the patient groups where conventional polyherbal wound therapies could be effective.

### 2.7. Data Retrieval and Synthesis

Titles and abstracts were screened by two independent reviewers. Potentially viable studies were then retrieved and reviewed in details according to the inclusion and exclusion criteria imposed on the entire text. In instances where it was impossible to extract or compute the effect sizes using the published data, the respective authors were approached through email to provide extra statistical data The discrepancies were decided by consensus or involving a third reviewer. Papers that were based on original clinical studies were only used. Every article that was found in the searches was imported to Microsoft Excel 19, and duplicates were eliminated. Initial screening procedures were done in the title and abstract reviews, and irrelevant articles were eliminated. The rest of the studies were then subjected to a critical screening by full‐text screenings. Inter‐reliability between the two reviewers during the title and abstract screening stage was assessed via Cohen′s kappa (*κ*) statistic to determine agreement beyond chance. The calculated *κ* value was 0.84, indicating strong agreement [23]. Any disagreements were resolved by consensus before proceeding to full‐text screening. Any oppositions were put to rest through consensus before full‐text screening was done. Articles that did not have a complete text and those that were considered irrelevant were excluded. To make the presentation of the findings more desirable, the data were systematized and presented in figures and select findings summarized in tables. The development of a data extraction form and pilot test was carried out. Three reviewers extracted the data of the studies included. Variables extracted were those related to the study (author [s], year of publication, country, study design, sample size, randomization method, and blinding), population (age, gender distribution, wound type, duration, severity, and baseline wound size), intervention (name of the polyherbal formulation, composition, form, number of herbs, route of administration, frequency, duration, and dosage), comparator (type of control: standard care, placebo, etc.), and outcomes (primary and secondary, measured, respectively, as wound‐healing time, wound‐healing rate, wound area reduction, c‐myc, and k6). Any differences in data extraction were solved by discussion.

### 2.8. Assessment of the Risk‐of‐Bias Quality

Three reviewers performed the evaluation of the risk of bias of each included study independently with the Cochrane Risk of Bias 2 (RoB 2) tool of randomized trials [24, 25]. The tool evaluates five domains (bias arising from the randomization process, bias due to deviations from intended interventions, bias due to missing outcome data, bias in measurement of the outcome, and bias in selection of the reported result). Each domain was judged as “low risk,” “some concerns,” or “high risk.” An overall risk‐of‐bias judgment was assigned accordingly. Disagreements were resolved by a third reviewer.

### 2.9. Statistical Analysis

When evidence of a sufficient level of homogeneity across study designs, interventions, and outcome measures became apparent, quantitative synthesis (meta‐analysis) was carried out using the online platform http://metaanalysisonline.com (https://http://metaanalysisonline.com; accessed August 12, 2025). The values of the sample, standard deviations (SDs), and mean of the results of the intervention and control group were then immediately typed into the application. Before the entry of data, standard errors (SEs) in each individual study were transformed into SDs. The continuous results were compared within a random‐effects model to achieve the expected methodological and biological heterogeneity. To cover the different measurement scales used, effects sizes were given as mean differences (MDs) with 95% confidence intervals (CIs). Between‐study variance was estimated by using the DerSimonian and Laird method of moments, and heterogeneity was measured by Cochran‐Q test and the *I* ^2^ statistic; *I* ^2^ exceeding 50% represented a significant level of heterogeneity. Publication bias was assessed both visually, using funnel plots, and statistically, using the regression test by Egger [26], which is executed on http://metaanalysisonline.com. The *p* value of less than 0.05 was taken as evidence of substantial asymmetry. The general strength of the evidence that supported each outcome was assessed based on Grading of Recommendations Assessment, Development, and Evaluation (GRADE) framework [27]. The areas measured were risk of bias, inconsistency, indirectness, imprecision, and publication bias. The evidence quality was then rated as high, moderate, low, and very low.

## 3. Results

### 3.1. Study Selection

The PRISMA flow diagram (Figure 1) illustrates the process of selection of the study. By searching electronic databases, 1340 records were retrieved at the cutoff of August 11, 2025, including 203 records in PubMed, 875 records in SCOPUS, 98 records in AMED, and 164 records in LILACS. After elimination of 232 duplicate records, the screening process was left with 1108 different records. At the title and abstract screening stage, 868 records were also excluded since they did not pass the inclusion criteria. The most common exclusion criteria were that the articles were reviews, measured nonclinical outcome, used single‐herb intervention (including in vitro or anial data), or did not have a comparative arm. As such, 240 records were sent to a more detailed consideration. Of these, 176 were filtered out as non‐English, irrelevant, or not available in full text after a thorough evaluation, and 64 records were eligible to be considered. A further 56 publications were eliminated after the completion of an eligibility poll as a result of not fulfilling the designated inclusion criteria: 20 lacked population, 12 failed to meet the intervention criteria, 14 lacked outcome data, and 10 were excluded due to the control criteria. Finally, the final systematic review included eight studies [28–35] that had met all preselected criteria of inclusion. These studies were located either by search of statistical data in published literature or by e‐mailing to the respective authors. The researches that did not have enough data to be included in the meta‐analysis were restricted to the qualitative synthesis part of the review. The latest article to be included in this list can be found in 2011, but half of the articles were published after 2015, which may suggest the presence of an increasing amount of evidence about the effectiveness of polyherbal preparations in the wound‐healing process.

### 3.2. Assessment of the Risk of Bias of the Included Studies

In order to determine the methodological quality of all the included studies, Cochrane RoB 2 tool of RCT was used. The assessment revealed a range of bias levels across domains such as bias arising from the randomization process, due to deviations from intended interventions and due to missing outcome data, in measurement of the outcome, and bias in selection of reported results. Some studies had methodological rigor, and some of them had significant weaknesses.

**Figure 2:**
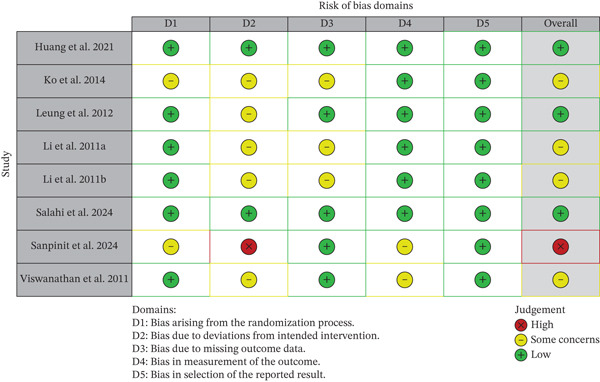
Analysis of risk of bias in the included clinical studies.

**Figure 3:**
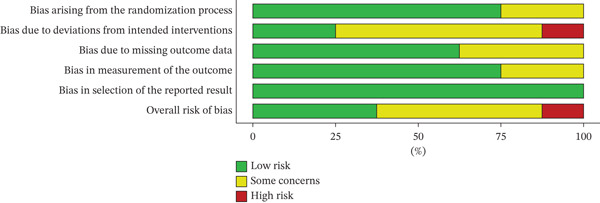
Summary of the risk of bias in the included clinical studies.

The risk‐of‐bias overview of the included studies is illustrated in Figure 2, and the overview of the risk of bias is provided in Figure 3 (based on Table S2: Risk assessment of studies included in investigating the clinical effectiveness of traditional polyherbal formulations for wound healing) as presented. These numbers will demonstrate how well reporting and bias measurement are performed using the RoB 2 tool in relation to the five domains. The quality of bias due to the randomization process was usually low; 75% of the studies (*n* = 6) did sufficient random sequence generation and allocation concealment [28, 30–33, 35]. On the other hand, 25% (*n* = 2) of the studies [29, 34] were concerned in this area meaning that they were not detailed enough or there may be deficiencies in the randomization. Prejudice based on deviations of the intended interventions had the highest variability. Although 62.5% of the studies (*n* = 5) were assessed to have some concern [29–31, 33], others, especially the Sanpinit et al. [24], were high as there were protocol deviations or absence of blinding which could affect the behavior or care of the participants. Missing outcome data bias was largely minimal (62.5%, *n* = 5), indicating that there were minimal attrition rates that were evenly distributed among the groups [28, 30, 33–35]. Nevertheless, 37.5% of the research papers [29, 31, 32] had certain issues, which might have been caused by the absence of full data processing. Most of the bias in the measurement of results were low (60%, *n* = 6), which indicated that the outcome assessors were usually blinded or the outcomes were measured objectively [28–33]. However, a quarter of the studies [34, 35] endeavored doubts the validity of the research, probably because of the use of subjective measures of outcomes without randomization. Bias in the selection of the reported result was generally low (100%, *n* = 8) [28–35]. The total risk‐of‐bias ratings were between low and high. High‐risk studies [34] often included flaws in randomization with inconsistency of considered interventions and issues with measuring outcomes. Huang et al. [28], Leung et al. [30], and Salahi et al. [33], on the contrary, showed a very low risk in domains, which gives more confidence to their results compared to Ko et al. [29], Li et al. [31], Li et al. [32], and Viswanathan et al. [35], which reported certain concerns.

### 3.3. Study Characteristics

**Figure 4:**
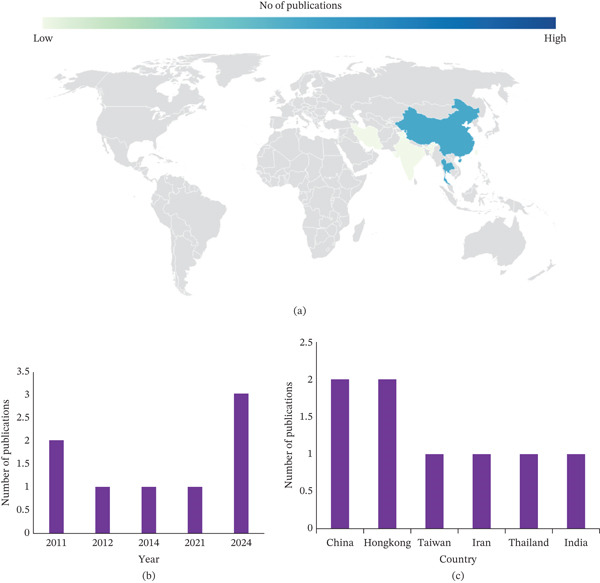
(A) Geographical distribution. (B) Number of publications per year of included studies reflecting the demographics of participants assessed for the clinical effectiveness of traditional polygenic formulations for wound healing. (C) Number of publications per country.

The general characteristics of the included studies are presented in Table 2 and Figure 4.

**Table 2:** General characteristics of the included studies.

Study	Year	Country	Sample size	Sample size per group	Study design	Randomization method	Blinding
Huang et al. [28]	2021	Taiwan	236	122/Group 1 114/Group 2	Randomized controlled trial, multicenter	Computer‐generated block randomization scheme	Evaluator blind
Ko et al. [29]	2014	Hong Kong	16	8/group	Randomized controlled trial	N/M	Double blind
Leung et al. [30]	2012	Hong Kong	80	40/group	Randomized placebo‐controlled clinical trial	Block randomization scheme	Double blind
Li et al. [31]	2011a	China	62	31/group	Randomized controlled trial, multicenter	Random number table	Single blind
Li et al. [32]	2011b	China	56	28/group	Randomized controlled trial, multicenter	Online tool—generation of randomization lists	Single blind
Salahi et al. [33]	2024	Iran	50	25/group	Randomized controlled trial	Shuffling sealed envelopes technique	Double blind
Sanpinit et al. [34]	2024	Thailand	56	28/group	Randomized controlled trial, multicenter	Sequential and random allocation	Double blind
Viswanathan et al. [35]	2011	India	40	20/group	Randomized controlled trial, unicenter	NM	NM

Abbreviation: NM, not mentioned.

### 3.4. Demographic Distribution

The studies were conducted in six countries, with Hong Kong and China as leading countries, with 33.33% of the studies (*n* = 2) [29–32], respectively, and with one study each from Thailand [34], Taiwan [28], Iran [33], and India [35]. The selected studies were published between 2011 and 2024. The years 2011 and 2024 each have two or more publications.

### 3.5. Randomization Technique and Blinding

The studies included were mainly RCT studies, but a mixture of single‐ and multicenter designs was used. The majority of the trials were parallel group. The sample size was quite different, as it was 16 participants [29] and 236 participants [28]. Most of the RCTs had an adequate description of randomization procedures. Li et al. [31] employed a random number table, Huang et al. [28] employed a computer‐generated block randomization scheme, and Leung et al. [30] employed a block randomization approach. Li et al. [32] created randomization lists through online tool, compared to Salahi et al. [33] in which shuffling sealed envelope technique was utilized. Sequential and random allocation was used by Sanpinit et al. [34]. But Ko et al. [29] and Viswanathan et al. [35] did not describe their procedures of randomization. Different studies had different blinding procedures. Four studies used the design of a double blind [  35, 29, 30, 34], so no participant and outcome assessors knew who was allocated into which group. Two studies reported single‐blind designs in which only one party (usually the evaluator) was blinded [30, 31]. Efforts by Huang et al. [28] involved the evaluation blinding alone. There was one study [35] that did not mention blinding or reported no blinding.

### 3.6. Sampling and Sampling Size

The included studies varied considerably in sample size, reflecting differences in study design and recruitment capacity. Large multicenter RCTs, such as Huang et al. [26], with 236 participants, and Li et al. [31], with 62 participants (31 per group), provided robust group comparisons. The moderate‐sized trials included Leung et al. [30], who had 80 participants (40 per group); Li et al. [32], who had 56 participants (28 per group); and Salahi et al. [33], who had 50 participants (25 per group). Smaller studies, such as Ko et al. [29], with 16 participants (eight per group), and Viswanathan et al. [35], with 40 participants (20 per group), contributed additional data.

### 3.7. Population Characteristics

Population characteristics such as age, sex (gender), duration of diabetes, wound location, duration of wounding, severity, and baseline wound size were quantitatively assessed. These characteristics are presented in Table S3 (Assessment of population characteristics in relation to clinical effectiveness of traditional polyherbal formulations for wound healing).

#### 3.7.1. Age

The median age of the study participants used in the analysis was between around 54 and 74 years, thus, indicating a middle‐aged to elderly group of population. The youngest participants were reported in Salahi et al. [33] (mean 55.6–57.9 years), and the oldest one was reported in Ko et al. [29] (mean 72.1–74.0 years). The majority of the studies involved adults between the age of 50 and 70, for example, Li et al. [31] (46.254.1 years), Li et al. [32] (60 ± 13 years), and Viswanathan et al. [35] (58.7–59.4 years). One of the trials covered wider age spans including Salahi et al. [33] (18–80 years); however, the values were still in the late 50s. This suggests that the clinical support of conventional polyherbal formulations in wound healing is based largely on older adults who are more susceptible to chronic wound since they have comorbidities like diabetes and vascular disease.

#### 3.7.2. Gender Distribution

The level of representation of both genders was not consistent in all the studies included, as most of the trials recruited more male subjects. In some of the studies, there was a distinct male dominance like Viswanathan et al. [35] who used 12 males and seven females in the polyherbal group and Li et al. [32] who reported that the intervention and control groups had a higher number of men as compared to women. Others were more balanced in terms of representation; for example, Li et al. [31] used 13 women and 18 men in the Chinese medicine group (CMG) and 15 women and 16 men in the Western medicine group (WMG), but in Ko et al. [29] the arms had equal numbers of men and women. Huang et al. [28] cited 61 and 175 females and males, respectively, with the same sex ratio between the groups. Overall, the aggregate data of pooled data indicate male dominance in majority study populations.

#### 3.7.3. Diabetes and Wound Duration

The duration of diabetes as reported by different studies was also heterogeneous according to the different profiles of patients. The average duration of the disease in various trials was around 6 years [31] to more than 13 years [35], with intermediate results of 7.2–13.4 years (29), 7.3–11.8 years (30), and about 8–12 years (31). Li et al. [31] gave a mean of about 9.5–10 years, and Salahi et al. [33] gave a mean of 11.0–12.8 years. Sanpinit et al. [34] found that there were significant group differences, the intervention participants had a mean age of 6.04, and the controls had a mean age of 10.28. In general, the majority of the respondents had a significant history of diabetes, usually more than 7–10 years, a factor that has been known to negatively influence wound‐healing outcomes. The study period of wounds was significantly different among the research participants. Li et al. [31] described 0.72 ± 0.65 years in the CMG and 0.41 years in the WMG, whereas Huang et al. [28] described 7.15 ± 13.4 months with majority of the patients having less than 6 months of wounds. Ko et al. [29] gave a range of durations of 56–105 days, and Leung et al. [30] gave 7.8 ± 8.2 and 12.9 ± 24.6 weeks of duration in the treatment and control groups, respectively. Li et al. [32] obtained a median of about 7 months, and Sanpinit et al. [34] gave 9.99 months in the treatment group and 4.88 months in the control group.

#### 3.7.4. Location, Severity, and Size of Wound

The location of the wounds used in the studies included was mostly the lower limb, which is consistent with the research interest in diabetic foot ulcers and the associated chronic wounds. Most of the studies such as Li et al. [31], Huang et al. [21], Li et al. [32], Salahi et al. [33], Sanpinit et al. [34], and Viswanathan et al. [35] focused on lower limb ulcers, frequently plantar or nonplantar. Ko et al. [29] and Leung et al. [30] outlined extensive distributions, toes, dorsal side of the foot, ankle, and foot heel being typical. Salahi et al. [33] have described injuries on the toes, soles, heels, back, and legs. Viswanathan et al. reported plantar forefoot, midfoot, and hindfoot ulcers [35]. All in all, the pattern of location of the wounds is linked with the neuropathic and ischemic complications of diabetes.

The extent of injuries in the participants was different in the studies with most of the trials using the Wagner grading system. The moderate and severe ulcers were present in many of the enrolled patients. As an example, Huang et al. [28] had only predominantly Wagner Grade 2 ulcer (184/236), whereas Li et al. [32] and Salahi et al. [33] used Grades 1–3, and the most common was Grade 2. Sanpinit et al. [34] comprised predominantly Grade 1 ulcer, whereas Viswanathan et al. [35] recorded more Grade 3 ulcers. Other researches, like Ko et al. [29], were concentrated on mild diabetic foot ulcers. This variability demonstrates a wide clinical range among the populations used. The size of the wound in the base was quite different between studies, as the nature of wounds and their severity is heterogeneous. The average area of the wound was between 0.33 cm^2^ (Ko et al. [2014] placebo group) and 28.7 ± 31.3 cm^2^ (Leung et al. [30] herbal treatment group [HTG]) with some studies showing moderate ulcers of 3–10 cm^2^. Li et al. [31] reported traditional and Western medicine wound size of 1654 and 1452 mm^2^, respectively. In certain research, sizes were stratified (e.g., < 5 vs. > 5 cm^2^), but other studies gave median or categorical values. The differences in the size of the first wound have significant prognostic effects, with bigger wounds usually linked to slower recovery. Therefore, the size of wounds is one of the main factors involved in the interpretation of comparative effectiveness among interventions.

### 3.8. Intervention Characteristics

As depicted in Table 3, intervention characteristics such as herbal composition, number of herbs that constitute the polyherbal mixture, form of polyherbal mixture, route of administration, dose, frequency, duration of treatment, use of placebo, and SWC were assessed and presented.

**Table 3:** Assessment of the intervention characteristics in relation to the clinical effectiveness of traditional polyherbal formulations for wound healing.

Author	Name of polyherbal	Composition	No of herbs	Form/route of admin.	Dose/frequency	Duration of treatment	Placebo	Standard wound care
Huang et al. [28]	ON101 cream	*Plectranthus amboinicus; Centella asiatica*	2	Cream base/topical	Fully cover the target ulcer, without exceeding 2 mm in thickness/twice daily	Up to 16 weeks	N/A	Both groups received standard wound care
Ko et al. [29]	NF3	*Astragali Radix; Radix Rehmanniae*	2	Powder containing extract granules/oral	10 g of NF3/once daily	6 months	A matching placebo was used, consisting of sodium carboxymethyl cellulose, with identical color and taste to NF3	All subjects, in both the NF3 and placebo groups, received standard care
Leung et al. [30]	Herbal formula	*Radix Astragali;* *Radix Rehmanniae;* *Rhizoma Atractylodis;* *Radix Stephaniae;* *Radix Polygoni;* *Rhizoma Smilacis;* *Poria;* *Rhizoma Dioscoreae;* *Fructus Schisandrae;* *Cortex Moutan;* *Fructus Corni;* *Rhizoma Alismatis;*	12	Granules/oral	N/M/twice a day	4 weeks	A placebo was used in the randomized controlled trial. The paper indicates that the control group received a placebo	All patients, including those in the herbal treatment and placebo groups, received standard treatment
Li et al. [31]	Hongyou ointment and Shengji powder	*Gypsum Fibrosum: hydrargyrum oxydatum crudum;* *Resina Draconis;* *Resina Olibanum;* *Dong pellet;* *Myrrh;* *Borneolum syntheticum*	7	Hongyou ointment: ointment/topical Shengji powder: powder	Hongyou ointment (1 g/cm^2^)/once a day Shengji powder (0.1 g/cm^2^)/once a day	CMG up to 4 weeks	N/A	WMG mupirocin ointment, growth factor (bFGF, 100 AU/cm^2^), and Vaseline gauze
Li et al. [32]	Tangzu Yuyang ointment (TYO)	*Coptis chinensis;* *Ligusticum chuanxiong;* *Atractylodes lancea;* *Panax notoginseng;* *Angelica sinensis;* *Arnebia euchroma;* *Phellodendron chinense;* *Rheum officinale;* *Borneolum syntheticum*	9	Ointment/topical	Based on the area of the ulcers, applied as a layer of approximately 1 mm thick/every 1 to 3 days	1 to 3 days or until complete closure of the ulcer or for 24 weeks	No separate placebo group	All patients in both the treatment and control groups received standard wound therapy (SWT)
Salahi et al. [33]	Dermaheal ointment	*Amebia euchroma;* Thymes; *Maticaria chamomilla; Curcuma longa;* olive oil	5	Ointment/topical	Entire surface of the ulcer, with an application thickness of approximately 1 mm/daily	Four consecutive weeks	A placebo ointment was used in the study. The placebo was primarily composed of Eucerin with the addition of Vaseline	All patients in both the Dermaheal and placebo groups received standard care
Sanpinit et al. [34]	Ya‐Samarn‐Phlae (YaSP) THR‐SK010	*Garcinia mangostana;* *Oryza sativa;* *Curcuma longa;* *Areca catechu;*	4	Infused oil/topical	2 mL of the oil per square centimeter of the wound area/daily	12 weeks	N/A	All patients in both groups received standard care
Viswanathan et al. [35]	Diabetic wound cream or polyherbal formulation cream	*Glycyrrhiza glabra;* *Musa paradisiaca;* *Curcuma longa; Pandanus odaratissimus;* *Aloe vera;* *Cocos nucifera oil*	6	Cream/topical	N/M/daily	5 months	N/A	The control group received silver sulphadiazine cream as the standard treatment

### 3.9. Number of Herbs in the Polyherbal Mixtures

In the studies included, the quantity of herbs used in polyherbal preparations was rather diverse and reflected variations in the conventional modes of preparation and therapeutic aims. Simpler preparations, which only contain two herbal constituents, were reported by Huang et al. [28] (ON101 cream: *Plectranthus amboinicus* and *Centella asiatica*) and Ko et al. [29] (NF3: *Astragali Radix* and *Radix Rehmanniae*). Four to seven herbs in moderately complex mixtures were found in half of the studies (50%) [31, 33–35]. Finer recipes that used a minimum of nine botanical ingredients were represented by Tangzu Yuyang ointment (nine herbs) in Li et al. [32] and a 12‐herb formula described by Leung et al. [30]. This theoretical variety highlights the heterogeneity of polyherbal wound‐healing interventions.

### 3.10. Form of Polyherbal Formulation

The reviewed studies used various classical polyherbal preparations, which were associated with local practices and curative goals. Topical application in the form of ointments, creams, or infused oils (e.g., Hongyou ointment, ON101 cream, Dermaheal, Tangzu Yuyang ointment, and Ya‐Samarn‐Phlae [YaSP] oil) was most frequently used to deliver the interventions, thus, providing direct effect to the wound bed (as was proven in Huang et al. [28], Li et al. [31], Li et al. [32], Salahi et al. [33], and Sanpinit et al. [34]). Oral granules, drinks, or powders (e.g., NF3, Leung herbal formula, and Wong herbal drinks) were used in three studies [29, 30, 35] with a purpose of systemic alteration of inflammatory processes and metabolic pathways. The heterogeneity of preparation and administration route is indicative of the holistic philosophy of traditional medicine. Whereas topical preparations tend to focus on wound‐site inflammation and wound tissue remodeling, oral preparations are supposed to stimulate the innate wound healing. Although different in forms, almost all the studies guaranteed the concurrent SWC, which allowed to provide a balanced evaluation of the added value of each formulation.

### 3.11. Route of Administration

Two routes of administration were used: topical (ointments, creams, oils, and powders) in six studies [28, 31–35] and oral (granules, powders, or herbal drinks) in two studies [29, 30]. The route of administration may influence pharmacokinetics and therapeutic outcomes; planned subgroup analyses will explore these differences.

### 3.12. Dose and Frequency of Administration

The doses of the polyherbal formulations varied by route and formulation type. Topical treatments such as Hongyou ointment (1 g/cm^2^) and Shengji powder (0.1 g/cm^2^) were applied once daily [31], whereas ON101 cream was applied twice daily for up to 16 weeks [28]. Similarly, Tangzu Yuyang ointment [32] was applied every 1–3 days in a 1‐mm layer. The oral preparations such as NF3 and herbal granules used were normally given as a single dose of 10 g a day [29] or twice a day [31, 35]. YaSP oil topically at 2 mL/cm^2^ daily was given [34]. Majorities of the studies adapted application in terms of wound size thereby keeping a regular daily, or twice‐daily frequency of application consistent across interventions, thus, depicting a standardized yet individualized dosing plan.

### 3.13. Duration of Treatment

The duration of polyherbal interventions varied substantially across the included studies, reflecting differences in wound chronicity, formulation type, and study design. Topically applied formulations such as ON101 cream and Dermaheal ointment [28, 33] were administered for up to 16 and 4 weeks, respectively, whereas oral polyherbal products such as NF3 and herbal drinks [29] were administered over 6 months and 5–30 weeks. Other interventions such as Hongyou ointment [31] and YaSP oil [34] came with a shorter duration of 4–12 weeks, respectively, depending on the studies. There were regimens with longer application up to the absolute closure of the ulcer, or a specific period of time (e.g., 24 weeks in Li et al. [32]). This variance was considered in the interpretation of outcome efficacy and during the subgroup analysis in order to determine time‐dependent therapeutic responses.

### 3.14. Placebo and SWC

Four of the included studies had placebo controls. The placebo used by Ko et al. [29] was sodium carboxymethyl cellulose which was set to look and taste like NF3 granules. Leung et al. [30] also employed the use of a placebo, but little has been stated about its composition. To simulate the texture of the active intervention, Salahi et al. [33] used a topical placebo ointment comprising Eucerin and Vaseline. Conversely, 60% of the studies [20, 31, 32, 34, 35] were not using a placebo; they compared it with the way the wound was managed normally or with other treatments that were on the go. Placebos were usually supplemented with normal care where they were used to treat patients ethically. These studies have well‐matched placebo groups that contribute to the internal validity.

In all the studies that were included, both in the control and intervention groups, SWC was provided, thus, making the studies comparable. SWC is often a combination of routine wound cleansing, debridement, infection management, offloading (in diabetic foot ulcers), and protective dressing. There were other studies like Li et al. [31] that used mupirocin ointment, Vaseline gauze, and recombinant basic fibroblast growth factor (bFGF). Silver sulfadiazine cream has been used by other researchers like Viswanathan et al. [35] as standard comparator. Some of the trials were conducted by Ko et al. [29], Leung et al. [30], and Sanpinit et al. [34] which focus on applying best practice guidelines to diabetic foot ulcers care. This standardization of SWC was used to remove the effects of polyherbal preparations and confounding. Therefore, any intervention that was included in it was assessed with evidence‐based traditional wound management practices.

### 3.15. Clinical Efficacy Parameters

Table 4 shows the clinical efficacy parameters used to measure the effectiveness of the polyherbal mixtures on wounds. The clinical efficacy parameters assessed quantitatively included the wound‐healing time, healing rate, healing and completely effective rates, wound area reduction, HbA1c levels, *β*‐catenin expression, and c‐myc and K6 expression levels.

**Table 4:** Assessment of clinical efficacy parameters in relation to the clinical effectiveness of traditional polyherbal formulations for wound healing.

Author	Wound‐healing time (days)	Healing rate (%)	Healed and completely effective rate (%)	Wound area reduction	*β*‐Catenin	c‐myc	K6	HbA1c level
Huang et al. [28]	ON101: 98 days Control: N/M	ON101: N/M Control: N/M	FAS ON101: 60.7 Control: 35.1 mITT ON101: 61.9 Control: 33.9	NM	N/M	N/M	N/M	Baseline: 8.1*%* ± 1.6*%* ON101: 8.6*%* ± 1.8*%* Control: 7.9*%* ± 1.6*%*
Ko et al. [29]	NF3: 125 days Placebo: 137 days	NF3: 3.55 Placebo: 1.52	NF3: 52.6 Placebo: 48.0	NF3: 47.8% Placebo: 14.1%	N/M	N/M	NF3 1.24‐fold Placebo N/M	Pretreatment NF3: 6.4*%* ± 0.7*%* Placebo: 6.7*%* ± 1.4*%* Post treatment NF3: 6.5*%* ± 0.7*%* Placebo: 6.4*%* ± 1.4*%*
Leung et al. [30]	HTG: 5.91*%* ± 1.36 Placebo: 9.15 ± 1.90	HTG: 85 Placebo: N/M	HTG: 85% Placebo: N/M	HTG: 28.7 ± 31.3 cm^2^ Placebo: 26.7 ± 27.3 cm^2^	N/M	N/M	N/M	N/M
Li et al. [31]	CMG: 22.71 ± 5.46 WMG: 26.56 ± 7.56	CMG: 33.33 WMG: 26.92	CMG: 81.48 WMG: 57.69	NM	After CM treatment 101.88 ± 10.76 Before treatment 140.42 ± 8.45	After CM treatment 113.27 ± 16.75 Before treatment 153.79 ± 8.32	After CM treatment 90.39 ± 11.07 Before treatment 151.29 ± 7.39	N/M
Li et al. [32]	TYO: 96 ± 56 SWT: 75 ± 53	4 weeks TYO: 16.7 SWT: 20.8 12 weeks TYO: 37.5 SWT: 33.3 24 weeks TYO: 79.2 SWT: 58.3	12 weeks TYO: 79.2 SWT: 41.7 24 weeks TYO: 91.7 SWT: 62.5	4 weeks TYO: 25% SWT: 25% 12 weeks TYO: 62.5% SWT: 37.5% 24 weeks TYO: 70.8% SWT: 41.7%	N/M	N/M	N/M	TYO: 9.9*%* ± 2.11*%* SWT: 8.8*%* ± 1.92*%*
Salahi et al. [33]	N/M	N/M	Dermaheal: 56 Placebo: 12	Both groups exhibited a decrease in ulcer size	N/M	N/M	N/M	Dermaheal: 8.3*%* ± 1.71*%* Placebo: 8.8*%* ± 2.47*%*
Sanpinit et al. [34]	N/M	N/M	YaSP: 76 Control: 16	YaSP: 2.66 ± 0.72 cm^2^ Control: 3.54 ± 0.78 cm^2^	N/M	N/M	N/M	YaSP: 7.95*%* ± 0.37*%* Control: 8.05*%* ± 0.47*%*
Viswanathan et al. [35]	Polyherbal: 43 days SSC: 43 days	N/M	N/M	At baseline Length Polyherbal: 4.98 ± 1.5 cm^2^ SSC: 4.3 ± 1.4 cm^2^ Width Polyherbal: 3.54 ± 1.4 cm^2^ SSC: 3.0 ± 0.98 cm^2^ At 5 months Length Polyherbal: 3.39 ± 1.8 cm^2^ SSC: 2.8 ± 2.0 cm^2^ Width Polyherbal: 2.38 ± 1.4 cm^2^ SSC: 1.9 ± 1.3 cm^2^	N/M	N/M	N/M	Polyherbal: 10.5*%* ± 1.58*%* SSC: 10.9*%* ± 1.5*%*

*Note:* All HbA1c values are expressed in percent (NGSP standard). Wound area values are expressed in square centimeters unless otherwise indicated.

Abbreviations: CMG, Chinese medicine group; FAS, full analysis set; HTG, herbal treatment group; mITT, modified intention‐to‐treat; NM/N/M, not measured/not reported; SSC, silver sulfadiazine cream; SWT, standard wound therapy; TYO, Tangzu Yuyang ointment; WMG, Western medicine group; YaSP, Ya‐Samarn‐Phlae oil.

### 3.16. Wound‐Healing Time (Days) and Healing Rate

The healing period of the wound differed significantly between researches, which is because wound type, severity, and intervention method are not the same. Li et al. [31] cited less duration of healing in the TCM group (22.71 ± 5.46 days) compared with the WMG (26.56 ± 7.56 days). Leung et al. [30] showed that the sample size of the HTG (5.91 ± 1.36) had much shorter time of healing in comparison with the placebo group (9.15 ± 1.90). According to Ko et al. [29], the time was decreased by 137–125 days on polyherbal therapy. Viswanathan et al. [35] found that the duration of healing was also the same, that is, 43 days in both the polyherbal and the silver sulfadiazine groups. The results of the study by Li et al. [32] and Huang et al. [28] were longer periods of recovery (75–98 days), as is characteristic of chronic diabetic foot ulcers. These results propose possible wound‐healing hastening due to polyherbal interventions though inconsistency emphasizes the value of wound nature and patient features.

### 3.17. Healed and Completely Effective Rates

The rate of clinical efficacy, which is a major indicator of its healing and complete effectiveness, differed among studies but tended toward polyherbal interventions. Li et al. [31] indicated that the complete effectiveness rate of the CMG (81.48%) was higher than the WMG (57.69%). According to Huang et al. [28], the number of patients who healed under ON101 treatment (60.7% FAS and 61.9% mITT) was significantly greater than the number of controls (35.1% and 33.9%, respectively). Similar results were observed by Ko et al. [29] as the NF3 and the placebo groups yielded similar results (52.6% vs. 48.0%). Leung and others [30] had high healing rates in polyherbal arms (85% and 87.5%, respectively) and few comparators. Li et al. [32] showed a steady increase, with a 91.7% healing rate in the TYO group after 24 weeks as compared to 62.5% of standard wound therapy.

### 3.18. Wound Area Reduction

The most common outcome is the wound area reduction that is a major measure of the clinical efficacy of polyherbal interventions. The findings of most of the studies showed a large decrease in wound size in cases of treatment with traditional polyherbal formulations as opposed to controls. As an illustration, Li et al. [31] found that the CMG decreased from 140.42 ± 8.45 mm^2^ to 101.88 ± 10.76 mm^2^. On the same note, Ko et al. [29] found that wound area reduction was 47.8% in the NF3 group compared to 14.1% in the placebo group. According to Sanpinit et al. [34], the mean reduction was found to be 55.8*%* ± 25.05*%*, and Leung et al. [30] found that in the polyherbal group the healing rates were 85%. This evidence indicates that polyherbal solutions are linked to significant wound contraction and tissue regeneration, and tend to be better than the standard or placebo intervention.

### 3.19. HbA1c Levels

Glycemic control (HbA1c) was inconsistently reported in all the studies included and acted as a valuable prognostic and outcome‐modifying variable in diabetic wound healing. Li et al. [31] also found that, after treatment using TCM, HbA1c levels significantly decreased, indicating that there was a systemic metabolic effect as opposed to local wound healing. Huang et al. [28] reported baseline HbA1c of 8.6*%* ± 1.8*%* (treatment group) and 7.9*%* ± 1.6*%* (control group), with minimal post‐treatment change. Ko et al. [29] observed stable post‐treatment HbA1c values (NF3: 6.5*%* ± 0.7*%*; Placebo: 6.4*%* ± 1.4*%*), indicating limited glycemic effect over 6 months. Leung et al. [30] and Viswanathan et al. [35] enrolled participants with higher baseline HbA1c (10.5% and 9.15%, respectively), reflecting more metabolically challenged populations. Overall, the heterogeneity in baseline and post‐treatment glycemic control across studies is likely to have substantially influenced wound‐healing responsiveness and should be carefully considered in the interpretation of pooled results.

### 3.20. *β*‐Catenin Expression and c‐myc and K6 Expression Levels

The main regulator in the Wnt signaling pathway to assess cell proliferation and epithelial regeneration, *β*‐catenin was considered a molecular marker of the wound‐healing efficacy in one of the studies included [31]. The expression of *β*‐catenin improved significantly and was considerably higher in the CMG after the treatment (101.88 ± 10.76) than it was at baseline (140.42 ± 8.45) which meant that *β*‐catenin upregulation was related to a faster tissue repair. The WMG, on the contrary, showed a less significant rise (153.79 ± 8.32 to 113.27 ± 16.75). These results indicate that polyherbal medication can improve wound healing through the regulation of Wnt/*β*‐catenin signaling. Although encouraging, the small number of studies that evaluate molecular markers should be taken with a grain of salt, and new studies should be conducted that combine the mechanistic outcomes. Very few studies incorporated in the research determined the molecular indicators of epidermal regeneration and cell proliferation. Li et al. [31] found that topical polyherbal treatment was associated with a significant decrease in the expression level of c‐myc and Keratin 6 (K6), which is a biomarker of hyperproliferative and poorly differentiating keratinocytes that get frequently upregulated in chronic wounds. In particular, the c‐myc level dropped to 113.27 ± 16.75 as compared to 153.79 ± 8.32 after treatment, and the K6 level dropped to 90.39 ± 11.07, compared to the level of 151.29 ± 7.39 before treatment. The implications of these cuts are the possibility of polyherbal intervention to stimulate wound healing through the normalization of the behavior of keratinocytes and the abnormal hyperplasia of the epithelial cell. These molecular marker data derive from a single study (Li et al. [31]) and should be considered preliminary and hypothesis generating only. The proposed mechanisms—including Wnt/*β*‐catenin signaling and keratinocyte normalization—require validation in independent studies across diverse polyherbal formulations and wound types before broader mechanistic conclusions can be drawn.

### 3.21. Other Related Outcomes

This review also examined secondary clinical outcomes across several included trials, specifically focusing on granulation time, amputation‐related data, and adverse effects, the results of which are presented in Table S4 (Assessment of clinical secondary outcomes in relation to clinical effectiveness of traditional polyherbal formulations for wound healing).

### 3.22. Granulation Time and Amputation‐Related Data

Granulation tissue formation is a vital indicator of wound‐healing progression. Only one study (10%), Leung et al. [30], reported granulation maturation time, which was measured as the duration before end‐stage skin grafting. To compare the two, both a Kaplan–Meier curve and a log‐rank test were used to test the time variance between the groups. By comparison, the other seven studies only provided quantitative values of total healing time, healing rate, and other similar values, which, although they cover components of the wound reparative process, such as the granulation phase, they do not give any specific granulation time.

The prevention of amputation is an ultimate outcome of chronic and infected wound treatment. In the chosen papers, a number of the recorded interventions were directed toward limb preservation. A comparison between complementary (CMG) and Western medicine cohort (WMG) was provided in detail by Li et al. [31]. Compared to the CMG, 30.7% (8/26) of participants in the WMG received surgical debridement which could be followed by amputation and could be an indication that the CMG could be beneficial in preventing surgical escalation. Encouraging limb salvage was reported by Leung et al. [30] of an HTG of 85% limb rescues with a placebo group. Salahi et al. [33] reported that 4% (1/25) of patients in the Dermaheal group and 12% (3/25) in the placebo group had amputations, respectively, which suggests a potential risk reduction with active treatment.

### 3.23. Adverse Effects

There was an inconsistent reporting of the safety profiles of the interventions studied. Li et al. [31] reported low rates of adverse events, and one event (3.7%) took place in the CMG. Data on adverse events were provided most comprehensively by Huang et al. [28]; treatment‐emerging adverse events (TEAEs) were prevalent in both groups: On101 group (45.9%; 52/122) and the comparator (52.6%; 60/114). Related TEAEs were not frequent (5.7% vs. 4.4%), and no serious TEAEs were present in either cohort. Li et al. [32] also found no differences in the overall rate of adverse events (29%) between TYO and SWT, but a greater rate of study‐related events in the SWT group (21% vs. 14%). Certain instances like infection and pain were similar in both groups with more serious adverse events and deaths in the TYO cohort. Salahi et al. [33] and Sanpinit et al. [34] did not mention any adverse effect, or they did not conduct safety evaluation. The same is done by Viswanathan et al. [35] who noted no significant adverse events. All in all, the rate of adverse effects was not high in the studies, and no salient safety issues were raised; yet, the incomplete reporting of adverse events in some trials does not allow a full safety evaluation.

### 3.24. Meta‐Analysis of the Clinical Effectiveness of Traditional Polyherbal Formulations for Wound Healing

This study also presents a detailed synthesis of the quantitative outcomes from studies investigating the clinical effectiveness of traditional polyherbal formulations for wound healing. The meta‐analysis focused on core physiological markers, including age (years), duration of diabetes, duration of wounding, baseline wound size, wound‐healing time, wound area reduction, and HbA1c. All analyses compared polyherbal formulation‐treated groups with appropriate controls, pooling data from studies that met the inclusion criterion of at least three comparable experimental models, as depicted in Figures S1 and S2 (Forest and funnel plot analysis showing the clinical effectiveness of traditional polyherbal formulations for wound healing compared to the control group).

The data on the average age of the respondents were obtained in seven studies [29–35], with 180 participants (experimental and control groups). The MD was aggregated using a random‐effects model with a weight of inverse variance. The MD across the age groups was 0.63 years (95*%*CI = −0.22 to 1.48) indicating no statistically significant difference in age between the intervention and control group. The overall effect test did not show any significant value and did not indicate the existence of heterogeneity (*I* ^2^ close to 0%) indicating a similar distribution of ages among the studies in both strength and direction. No asymmetry was detected in the funnel plot, and the regression (intercept = 0.36, 95*%*CI = −0.64 to 1.37, *p* = 0.511) by Egger proved the lack of publication bias. This baseline age balance makes the probability of age differences to confound observed treatment effects less likely. There were six studies (*n* = 149 in each arm) which reported mean diabetes duration [29, 30, 32–35]. The meta‐analysis also provided an MD of −2.16 years (95*%*CI = −4.75 to 0.42), which is slightly positive and statistically not significant in favor of the intervention group. The heterogeneity was high (*I* ^2^ = 70.5*%*, *p* < 0.0046) indicative of the duration of diabetes varying considerably across trials, both in magnitude and direction of effect. The possible causes involve the variation of recruitment patterns, population characteristics, and severity of diabetic complications. In spite of this heterogeneity, funnel plot symmetry and the Egger test (intercept = 1.53, 95*%*CI = −0.10 to 3.16, *p* = 0.139) did not indicate the presence of publication bias. The heterogeneity in the duration of diabetes can serve as a critically important modifier of wound‐healing patient results, and these considerations should provide considerable importance in the explanation of the effect estimates.

There were five studies with144 participants in each of these studies, and mean wound time before intervention was reported [30, 31, 33–35]. The MD of the polyherbal and control groups was 0.49 months (95*%*CI = −5.50 to 6.47), and the baseline difference between the two groups was not statistically significant. The degree of heterogeneity was high (*I* ^2^ = 97.8*%*, *p* = 0.0001), as there was a significant difference in wound chronicity among studies. This heterogeneity can be explained by different inclusion criteria, as some of the trials were concentrated on long‐term ulcers, and some included both acute and chronic lesions. The use of funnel plot analysis and Egger test (intercept = 2.13, 95*%*CI = −6.72 to 10.97, *p* = 0.670) did not prove that there was a publication bias. The large variability in the duration of the baseline wound may have an impact on the responsiveness to polyherbal interventions. Three studies (*n* = 93/group) provided data on initial wound size [30, 33, 34]. The MD of pooled was −0.63 cm^2^ (95*%*CI = −1.79 to 0.53) which has a statistically significant difference in favor of the control group. The heterogeneity was insignificant, and the assumption is that there was a similar size of baseline wounds across the studies included in the analysis. This balance improves the confidence that further variations in wound‐healing rates could not be explained by imbalance in the size of baselines. The analysis of funnel plot and the test reported by Egger (intercept = 1.03, 95*%*CI = −1.24 to 3.29, *p* = 0.537) did not indicate any sign of publication bias.

The time to wound healing was investigated in three studies (*n* = 99 in each group). The meta‐analysis resulted in the generation of an MD of −3.28 days (95*%*CI = −8.56 to 2.01), which offered an insignificant tendency toward faster healing in the intervention group [30–32]. The heterogeneity was minimal, which means that the estimates of effects were uniform between studies both in magnitude and in direction. Although the difference did not reach statistical significance, the small but consistent trend could be clinically relevant, especially if larger studies confirm the effect. No small‐study effects or publication bias was suggested by funnel plot and Egger (intercept = 0.88, 95*%*CI = −1.19 to 2.96, *p* = 0.557). Three studies (*n* = 73 per arm) found variation in HbA1c, which is an index of glycemic control [32, 33, 35]. The combined MD was −5.97% (95*%*CI = −30.86 to 18.93), and this was not statistically significant. The heterogeneity was severe (*I* ^2^ = 98*%*, *p* < 0.01), indicating a significant dissimilarity in the establishment of glycemic control, time of treatment, and simultaneous antidiabetic managerial practices. The large *I* ^2^ indicates that 98% of the study variation is due to actual heterogeneity and not due to random variation. Although HbA1c is not a wound‐healing measure, the inhibited glycemic control is a recognized barrier to wound healing; as such, baseline HbA1 level or postintervention HbA1 level could be an effect modifier that should be investigated further. No publication bias was suggested by funnel plot and Egger (intercept = −14.35, 95*%*CI = −20.12 to 8.58, *p* = 0.129).

In all the outcome and baseline variables examined, no statistically significant differences were found between the polyherbal and control groups. The alignment of the majority of the characteristics of the baseline, such as age and wound size, was quite satisfactory; the time of diabetes and wound duration had a high heterogeneity, which highlights variability within the study groups. Healing time of wounds showed a slight yet recurrent, nonsignificant tendency to improve in the polyherbal group of patients. Significantly, no pooled analysis showed that there was publication bias judged by the funnel plots or Egger tests. Lack of statistical significance in the pooled findings does not rule out a clinical benefit; some of the trials included were underpowered to capture small effects, and the diversity of formulation composition, dosage, route of administration, and extent of treatment may hush effects that would have been observed in a meta‐analytic framework. Evidence certainty in outcomes showed varying levels of evidence as given by the GRADE assessment (see Table S5: GRADE table for summary of findings based on meta‐analysis of studies on clinical effectiveness of traditional polyherbal formulations for wound healing). Baseline age had high certainty, with no group difference and little heterogeneity. Duration of diabetes had a low level of certainty as there was a significant level of heterogeneity (*I* ^2^ = 71*%*). The length of wound had very low certainty, which represents very large heterogeneity (*I* ^2^ = 98*%*) and high confidence limits. Baseline wound size and wound‐healing time were rated as moderate certainty; these both had consistent effects, but the small sample sizes dampened preciseness. The size of the HbA1c decrease was judged with a very low level of certainty, which is explained by a very high level of heterogeneity (*I* ^2^ = 98*%*) and the nondirect effect of the wound healing.

## 4. Discussion

This systematic review and meta‐analysis synthesized evidence in the form of RCTs on the clinical effectiveness of the traditional polyherbal preparations in wound management in the heterogenous population and etiology of wound. The findings have provided substantive information on the possible potential of these formulations as adjuncts to the traditional wound care, as well as clarifying the relevant methodological and contextual limitations that need to be considered in the future studies. Internal validity of the aggregated analyses is supported by the harmonization of the baseline characteristics of the investigations included. In this regard, the age, the size of the wound, years of diabetes, and chronicity of the wound (important prognostic factors influencing healing patterns) were widely balanced between the control and intervention groups and in this way alleviated the risk of selection bias [36].

It has been well established that age is a determinant of wound healing due to the age‐induced changes in immune functionality, angiogenic potential, and collagen production [37]. Our pooled analysis revealed no significant age difference between groups, suggesting that age‐related healing variability is unlikely to have influenced comparative outcomes. Also, the baseline wound size, which is highly related to the healing time and the likelihood of healing [38], did not have any significant difference between the groups, which further supported the validity of the aggregated healing events. However, chronicity of the wound and the number of years of diabetes show a substantial heterogeneity (*I* ^2^ values > 70%), indicating the presence of variability in the burden of the disease and chronicity. Chronic wounds are not similar to acute wounds; in most cases, they become characterized by incessant inflammation process, fibroblast senescence, and loss of muscle migration of keratinocytes [39, 40]. Variations in these factors across studies may have attenuated or obscured the true treatment effects, underscoring the importance of stratifying participants by chronicity in future trials. The heterogeneity observed in several meta‐analytic outcomes can be meaningfully linked to the risk‐of‐bias profiles of the included studies. Studies with high or unclear risk in the randomization domain (Ko et al. [29]; Viswanathan et al. [35]) and those with performance bias attributable to incomplete blinding (Sanpinit et al. [34]) introduced variability in both the direction and magnitude of treatment effects, particularly for subjective and clinician‐assessed outcomes. The extreme heterogeneity in HbA1c (*I* ^2^ = 98*%*) likely reflects not only true clinical variability in baseline glycemic control and concurrent antidiabetic management, but also methodological inconsistencies in outcome assessment, follow‐up duration, and reporting completeness factors closely tied to the identified risk‐of‐bias domains. Conversely, outcomes with lower heterogeneity, such as baseline age and wound size (*I* ^2^ ≈ 0*%*), correspond to studies with consistently low risk of bias in the randomization and outcome measurement domains. This correspondence underscores the critical role of methodological rigor in future trials for reducing unexplained heterogeneity in pooled analyses. In light of the substantial clinical heterogeneity observed encompassing differences in herbal composition, number of constituents (2–12 herbs), route of administration, treatment duration, and wound severity (Wagner Grades 1–3), the pooled effect estimates should be interpreted as exploratory rather than definitive. Subgroup analyses stratified by administration route and wound type, and sensitivity analyses excluding high risk‐of‐bias studies, are strongly recommended in future reviews as the evidence base expands.

The combined outcomes of the wound‐healing rate showed a trend toward higher rates in polyherbal formulations although statistical significance was not consistently achieved. This finding supports priori findings that a number of herbal constituents in polyherbal preparations have bioactive phytochemicals with prohealing activity, including curcumin in *Curcuma longa*, asiaticoside in *Centella asiatica*, and neem extracts in *Azadirachta indica* [41]. These bioactive compounds regulate various pathways involved in wound repair, including anti‐inflammatory effects, stimulation of fibroblast growth, and enhancement of angiogenesis [42]. The time to complete wound healing showed a nonsignificant mean reduction of approximately 3 days in the intervention groups; although of limited significance, the decrease has the potential of being clinically significant by reducing the risk of infection, costs of treatment, and morbidity in patients [43]. These lack statistical significance, which can be reasonably explained by small sample sizes and the fact that the duration of intervention was not constant, so that properly powered RCTs could help understand whether these effects can be reproducible. Glycemic control is an adjoining factor of healing in the case of diabetic wounds [44]. Ineffective glycemic control weakens the roles of leukocytes, cross‐linking of collagen, and angiogenesis that are involved in the development of chronic wound pathology [45]. The pooled analysis failed to indicate any significant effect of polyherbal preparations on HbA1c; the heterogeneity was too high (*I* ^2^ > 90*%*), and the baseline HbA1c and current antidiabetic medications and being frequently short in duration caused this heterogeneity. It is worth noting that some of the herbs used in the formulations like Gymnema sylvestre and Trigonella foenum‐graecum have been shown to have hypoglycemic effects [46], but their impact may require longer durations or standardized dosages to manifest in clinical outcomes.

A small number of the trials reported molecular markers such as c‐myc and K6 which are markers of the proliferation and differentiation status of keratinocytes. As an example, Li et al. [31] have reported that c‐myc and K6 were considerably decreased by polyherbal treatment, indicating that there was normalization of epithelial proliferation and differentiation. Mechanical plausibility of these results is that chronic wounds often have hyperproliferative (companied by impaired differentiation) epidermis [47]. While the molecular data from Li et al. [31] offer intriguing preliminary mechanistic insights into possible keratinocyte normalization via c‐myc and K6 downregulation, and Wnt/*β*‐catenin pathway activation, it is critical to emphasize that these findings originate from a single study with a relatively small sample. No other included trial reported molecular endpoints. These findings are therefore strictly exploratory and hypothesis generating, and should not be interpreted as evidence of a class effect for polyherbal formulations. Adequately powered mechanistic studies are needed before such molecular pathways can be confidently attributed to polyherbal wound interventions.

The meta‐analyzed evidence did not show any statistically significant variations in intervention and control groups in regard to major baseline variables, such as age, length of diabetes, wound duration, and wound size. This indicates that the incorporated researches were generally balanced and without a serious selection bias with regard to such features, hence, supporting the validity of the comparisons [36]. The current meta‐analysis has not determined a body of consistent evidence that polyherbal preparations with traditionally high polyherbal content are more effective in wound healing than in wound control; one pooled comparison showed a statistically significant result, but it is limited by the small size of studies, large interstudy heterogeneity in multiple analyses, and wide interventions and outcome variability.

Baseline age did not show significant intergroup difference, and heterogeneity was also insignificant, meaning that age‐based difference in wound‐healing ability [37] is unlikely to have played a role. Similarly, there were no significant differences in the baseline wound size between the groups, which is significant as a larger wound size normally requires more time to heal [38]. Thus, differences in the outcome of subsequent healing can be more likely explained by the intervention, and not by the fact that the sizes were different in the first place. Two baseline variables, on the other hand, duration of diabetes and a wound duration, showed a significant measure of heterogeneity (*I* ^2^ = 71*%*and 98*%* accordingly). The long‐term diabetes is accompanied by an impaired angiogenesis, neuropathy, and the dysfunction of the microvasculature, all of which slow wound healing [33]. On the same note, chronic wounds have a higher propensity to contain persistent biofilms, chronic inflammations, and cellular maladaptation [40]. Interstudy differences in these prognostic variables might confound or dampen any possible benefits of polyherbal formulations, and, in particular, where subgroup analyses are not feasible because of small sample sizes. In terms of time to wound healing, the tendency was not significantly different in support of polyherbal formulations of low heterogeneity. Though nonsignificant, the overall direction of effect is similar across the studies and points to a slight benefit which could prove clinically important in larger, well‐powered trials to the extent that even small decreases in the healing time can counteract infection risk, hospitalization, and treatment expenses [43].

Variations in HbA1c were also analyzed, as it is validated that glycemic control and wound‐healing outcomes have a strong relationship [44]. The pooled MD (−5.97%; 95*%*CI = −30.86 to 18.93) was not significant, and heterogeneity was extremely high (*I* ^2^ = 98*%*). In this way, the effect of polyherbal preparations on glycemic control is unclear and probably affected by the variations in baseline HbA1c, associated antidiabetic drugs, and intervention periods. Because glycemic control leads to poor leukocyte functioning, collagen synthesis, and angiogenesis [45], its optimization is a keystone of diabetic wound care, regardless of any adjunctive use. Interestingly, none of the pooled analyses showed any indication of publication bias, measured either by funnel plots or by the Egger test, further increasing the confidence that the provided data can provide an unbiased estimate of effects. However, the widespread lack of statistical significance of most of the outcomes does not always mean that they cannot work. Some of the trials that were included were small and could be underpowered to identify significant yet small clinic effects. Small powered studies may produce large confidence ranges and increase the probability of Type II error, particularly in multifactorial physiological processes like wound healing, which are multifactorial [48]. The potential mechanisms by which polyherbal formulations promote wound healing may be understood within the broader landscape of biologically active wound‐healing strategies currently under investigation. For instance, pH modulation at the wound site including the role of lactic acid in promoting epithelial repair and keratinocyte migration has been highlighted as a clinically relevant biochemical target, as demonstrated in a recent systematic review of episiotomy wound healing [49]. Similarly, platelet‐rich plasma (PRP), which concentrates autologous growth factors including PDGF, TGF‐*β*, and VEGF, has been evaluated as a biologically active adjunct in surgical wound settings, with randomized evidence demonstrating beneficial effects on postcaesarean scar healing [50]. Polyherbal formulations share conceptual overlaps with these approaches in their provision of multitarget bioactive modulation through anti‐inflammatory, angiogenic, and tissue‐remodeling phytochemicals. However, unlike PRP or defined pharmacological agents, the inherent variability in polyherbal composition and phytochemical standardization represents a key challenge in achieving comparable mechanistic clarity and translational consistency. Future research integrating standardized phytochemical profiling with wound biomarker assessments would help bridge this translational gap.

Moreover, there was significant discrepancy in the polyherbal formulations that included herb species, phytochemical standardization, dosage, and route of administration. This variability was probably due to this heterogeneity. Ayurveda and TCM are commonly used forms of traditional medicine that base prescriptions on patient constitution and wound properties [51], and are a real‐world practice, but are less synthesizable with modern evidence. In light of this substantial clinical heterogeneity encompassing differences in herbal composition, number of constituents (2–12 herbs), route of administration (topical vs. oral), treatment duration (4 weeks–6 months), and wound severity (Wagner Grades 1–3), the pooled effect estimates should be interpreted as exploratory rather than definitive. Prespecified subgroup analyses comparing topical versus oral routes of administration, and sensitivity analyses restricted to studies with low risk of bias or to diabetic foot ulcers exclusively, were planned but could not be executed meaningfully: Only three studies contributed data to each of the wound‐healing time and HbA1c pooled analyses, rendering any subgroup an analysis of one or two studies insufficient for interpretable inference. These analyses are designated as priority objectives for future updated systematic reviews as the evidence base grows. This could improve the reproducibility of future studies by standardization of herbal composition and quality control by phytochemical profiling. The duration of treatment was also extremely varied, between several weeks and several months; chronic wounds can be chronically treated and may need many weeks of therapy to fully epithelialize, and abbreviated treatment periods can be missing the optimum effect of the treatment. Furthermore, some of the trials contain a risk of performance bias due to the inconsistency in the reports on adherence to intervention protocols [24]. The bulk of the included studies used polyherbal formulations with conventional wound care, which would be the best practice, but may dilute the marginal benefit of the intervention that is observed in the controlled environment. However, such a pragmatic stance reflects the clinical reality that polyherbal compounds are normally used as adjunct forms of treatment and not as monotherapy.

Overall, the results obtained in this meta‐analysis can be compared to the results of the previous reviews of herbal and polyherbal treatments of wounds that showed promising but not strong outcomes in general because of methodological shortcomings and heterogeneity [52]. Our results extend the literature by quantifying the pooled effects of traditional polyherbal formulations specifically and providing a clearer picture of their current evidence base. The implications for practice and research from the studies are clear. Despite the absence of statistically significant effects on some of the outcomes, the observed trends, especially in terms of wound‐healing time, suggest potential benefits that warrant further investigation. Future RCTs can prioritize adequate sample sizes that are powered for clinically relevant differences; standardized and well‐characterized polyherbal formulations; longer follow‐up periods to ensure complete healing and recurrence rates; stratification or adjustment for key prognostic factors such as the wound duration, diabetes control, infection status, and the use of validated; and objective outcome measures, including histopathological and biochemical end points.

## 5. Limitations

There are a number of shortcomings that should be taken into account when interpreting these findings. The trials involved were very diverse in the polyherbal formulations, dosages, treatment periods, and types of wounds, thus, creating a heterogeneity between the analyses. Most of the researches used small sample sizes, which constrained the statistical power of the studies and accuracy of effect estimates. There was a high tendency of incompleteness of methodology reporting, especially on randomization procedures, allocation concealment, and blinding, which enhanced the risk of biasness. Also, inconsistency in outcome measure and time of follow‐up hindered direct comparison of studies. The majority of trials were held in particular Asian environments, which might not have allowed generalizing the results to other population groups, and the risk of publication bias cannot be excluded due to a small amount of eligible RCTs per outcome. Analytical approaches also varied across included trials: Only Huang et al. [28] explicitly reported intent‐to‐treat (ITT) analyses (full analysis set and modified ITT populations), whereas the remaining studies employed per‐protocol or completer‐based analyses. This variability in analytical population definitions may have introduced differential attrition bias, reducing the comparability of reported outcomes across studies and potentially inflating observed treatment effects in completer‐only analyses. We had anticipated conducting subgroup analyses comparing topical versus oral routes of administration and sensitivity analyses restricted to studies with low risk of bias. However, the limited number of included studies (*n* = 8 overall, with only three studies per pooled outcome for wound‐healing time and HbA1c) precluded such analyses, as any resulting subgroup would contain insufficient studies to yield meaningful or interpretable estimates. Future systematic reviews, as the evidence base grows, should prioritize these subgroup comparisons. Additionally, restricting pooled analyses to diabetic foot ulcers specifically would improve clinical homogeneity but was similarly not feasible within the current dataset given the overlap in wound etiology across trials.

A further methodological limitation is the restriction of study identification to four electronic databases (PubMed, Scopus, AMED, and LILACS) without supplementary grey literature searches (e.g., OpenGrey, ProQuest Dissertations and Theses, and ClinicalTrials.gov) or hand‐searching of reference lists, conference proceedings, or relevant journal issues. This decision may introduce retrieval bias by excluding unpublished studies, dissertations, or trial reports not indexed in the selected databases. Given the known positive‐result publication bias in complementary medicine research, the absence of grey literature searches may inflate apparent effect sizes, and this must be considered when interpreting pooled estimates. Future systematic reviews in this area should incorporate comprehensive search strategies, including grey literature and prospective trial registries, to address this limitation. Collectively, these limitations highlight the need for larger, rigorously designed RCTs that employ standardized formulations, clear outcome definitions, and transparent reporting practices.

## 6. Conclusion

This systematic review and meta‐analysis synthesized evidence from eight RCTs investigating the clinical effectiveness of traditional polyherbal formulations for wound healing, with particular relevance to diabetic patients. Pooled analyses of wound‐healing time and HbA1c reduction did not yield statistically significant results, and heterogeneity was extreme for HbA1c (*I* ^2^ = 98*%*), severely limiting confidence in effect estimates. Certainty of evidence, rated using the GRADE framework, ranged from high (baseline age comparability) to very low (HbA1c reduction and wound duration), underscoring the substantial uncertainty inherent in the current evidence base. While directional trends toward faster wound healing and greater wound area reduction were observed across formulations of diverse composition (2–12 herbal constituents) and routes of administration (topical and oral), these trends remained nonsignificant in pooled analyses, likely attributable to small sample sizes, substantial clinical heterogeneity, and methodological variability across trials. These findings indicate that polyherbal formulations cannot yet be recommended as evidence‐based alternatives to SWC. However, their potential as adjunctive strategies particularly given their multitarget bioactive mechanisms and cultural acceptability warrants further investigation through adequately powered, methodologically rigorous RCTs using standardized formulations, validated outcome measures, and sufficient follow‐up durations. Prespecified subgroup analyses by route of administration and wound type, and sensitivity analyses restricted to low‐risk‐of‐bias studies, should be incorporated into future evidence syntheses as the trial evidence base grows.

## Author Contributions

Samuel Abiodun Kehinde drafted the manuscript. Samuel Abiodun Kehinde, Nurulhusna Awaeloh, and Siriwan Kantisin conducted a literature search. Nurulhusna Awaeloh and Samuel Abiodun Kehinde independently screened the potential studies and extracted the data. Samuel Abiodun Kehinde and Sasitorn Chusri edited the manuscript. Sasitorn Chusri and Pinanong Na‐Phatthalung arbitrated any disagreements and ensured that no errors occurred during the review. All authors critically reviewed, revised, and approved the subsequent and final version.

## Funding

The article processing charge (APC) is supported by Mae Fah Luang University, Thailand. This research received financial support from the "National Science, Research, and Innovation Fund (NSRF)" (Fundamental Fund Grant No. 682A05005) and the Postdoctoral Fellowship Fund from Mae Fah Luang University, Thailand (Contract No. 10/2025).

## Disclosure

All authors have read and approved the final manuscript and agree to be accountable for its contents.

## Ethics Statement

This manuscript is a systematic review and does not present any negative impact issues. All operations adhered to ethical standards.

## Conflicts of Interest

The authors declare no conflicts of interest.

## Supporting information


**Supporting Information** Additional supporting information can be found online in the Supporting Information section. Additional supporting information can be found online in the Supporting Information section. **Supporting Information 1**. Table S1: Database search strategy for clinical studies investigating the clinical effectiveness of traditional polyherbal formulations for wound healing. **Supporting Information 2**. Table S2: Risk assessment of studies included in investigating the clinical effectiveness of traditional polyherbal formulations for wound healing. **Supporting Information 3**. Table S3: Assessment of population characteristics in relation to clinical effectiveness of traditional polyherbal formulations for wound healing. **Supporting Information 4**. Table S4: Assessment of clinical secondary outcomes in relation to clinical effectiveness of traditional polyherbal formulations for wound healing. **Supporting Information 5**. Figure S1: Forest plot analysis showing the clinical effectiveness of traditional polyherbal formulations for wound healing compared to the control group. **Supporting Information 6**. Figure S2: Funnel plot analysis showing the clinical effectiveness of traditional polyherbal formulations for wound healing compared to the control group. **Supporting Information 7**. Table S5: GRADE table for summary of findings based on meta‐analysis of studies on clinical effectiveness of traditional polyherbal formulations for wound healing.

## Data Availability

All data used in this work are found in the manuscript.
