# Supplementary material for: Clinical Effectiveness of Traditional Polyherbal Formulations for Wound Healing: A Systematic Review and Meta‐Analysis
Source: ScientificWorldJournal. 2026 May 11;2026:1551004. doi: 10.1155/tswj/1551004 (PMC13158697; doi:10.1155/tswj/1551004)
Supplement: Supplementary file 1 — Supporting Information Additional supporting information can be found online in the Supporting Information section. Additional supporting information can be found online in the Supporting Information section. Supporting Information 1. Table S1: Database search strategy for clinical studies investigating the clinical effectiveness of traditional polyherbal formulations for wound healing. Supporting Information 2. Table S2: Risk assessment of studies included in investigating the clinical effectiveness of traditional polyherbal formulations for wound healing. Supporting Information 3. Table S3: Assessment of population characteristics in relation to clinical effectiveness of traditional polyherbal formulations for wound healing. Supporting Information 4. Table S4: Assessment of clinical secondary outcomes in relation to clinical effectiveness of traditional polyherbal formulations for wound healing. Supporting Information 5. Figure S1: Forest plot analysis showing the clinical effectiveness of traditional polyherbal formulations for wound healing compared to the control group. Supporting Information 6. Figure S2: Funnel plot analysis showing the clinical effectiveness of traditional polyherbal formulations for wound healing compared to the control group. Supporting Information 7. Table S5: GRADE table for summary of findings based on meta‐analysis of studies on clinical effectiveness of traditional polyherbal formulations for wound healing. [file TSWJ-2026-1551004-s001.docx]

**Supplementary Data**

**Supplementary Table 1:** Database search strategy for *Clinical studies* investigating the Clinical Effectiveness of Traditional Polyherbal Formulations for Wound Healing

| Database | Keywords | Results |
| --- | --- | --- |
| PUBMED  12/08/2025 | KS-1: ("Wound Healing"[MeSH] OR "chronic wounds" OR "ulcer healing" OR "wound repair") AND ("polyherbal formulation" OR "compound herbal preparation" OR "multi-herbal" OR "traditional herbal formula") AND ("clinical trial"[Publication Type] OR "randomized controlled trial" OR "clinical effectiveness")  KS -2 : ("Wound Healing"[MeSH]) AND ("polyherbal" OR "multi-herbal") AND ("Traditional Chinese Medicine" OR "Ayurvedic Medicine" OR "Unani Medicine" OR "Traditional Medicine"[MeSH])  KS-3: ("polyherbal formulation" OR "Ayurvedic preparation" OR "traditional herbal formula") AND ("Wound Healing"[MeSH]) AND ("Treatment Outcome"[MeSH] OR "Therapeutic Effectiveness" OR "Healing Rate")  KS-4: 4. ("Herbal Medicine"[MeSH] OR "Plant Extracts"[MeSH] OR "Medicinal Plants"[MeSH]) AND ("Wound Healing"[MeSH]) AND ("Clinical Trial" OR "Effectiveness") | 4  10  4  185 |
| SCOPUS  12/08/2025 | KL-1: TITLE-ABS-KEY("wound healing" OR "chronic wounds" OR "ulcer healing" OR "wound repair") AND TITLE-ABS-KEY("polyherbal formulation" OR "multi-herbal" OR "traditional herbal formula") AND TITLE-ABS-KEY("clinical trial" OR "randomized controlled trial" OR "clinical effectiveness")  KL-2: TITLE-ABS-KEY("wound healing") AND TITLE-ABS-KEY("polyherbal" OR "multi-herbal") AND TITLE-ABS-KEY("Traditional Chinese Medicine" OR "Ayurvedic Medicine" OR "Unani Medicine")  KL-3: TITLE-ABS-KEY("polyherbal formulation" OR "Ayurvedic preparation" OR "herbal formula") AND TITLE-ABS-KEY("wound healing") AND TITLE-ABS-KEY("treatment outcome" OR "clinical outcome" OR "healing rate")  KL-4: TITLE-ABS-KEY("herbal medicine" OR "medicinal plant" OR "plant extract") AND TITLE-ABS-KEY("wound healing") AND TITLE-ABS-KEY("clinical trial" OR "effectiveness") | 4  4  6  861 |
| LILACS  12/08/2025 | KL-1: ("Wound Healing" OR "Chronic Wounds" OR "Skin Ulcer") AND ("Herbal Formulations" OR "Compound Herbal Preparations") AND ("Clinical Trials" OR "Clinical Efficacy")    KL-2: ("Wound Healing") AND ("Traditional Phytotherapy") AND ("Traditional Chinese Medicine" OR "Ayurveda" OR "Unani")  KL-3: ("Phytotherapeutic Preparations" OR "Ayurvedic Formulations") AND ("Wound Healing") AND ("Treatment Outcome" OR "Therapeutic Efficacy")  KL-4: ("Medicinal Plants" OR "Plant Extracts") AND ("Wound Healing") AND ("Clinical Trials" OR "Clinical Efficacy") | 17  61  6  80 |
| AMED  12/08/2025 | KL-1: (wound healing OR chronic wounds OR ulcer healing) AND (polyherbal formulation OR multi-herbal OR traditional herbal formula) AND (clinical trial OR clinical effectiveness OR randomized controlled trial)  KL-2: (wound healing) AND (polyherbal OR multi-herbal) AND (traditional medicine OR Ayurvedic OR Traditional Chinese Medicine OR Unani)  KL-3: (polyherbal OR Ayurvedic OR traditional herbal formula) AND (wound healing) AND (treatment outcome OR therapeutic benefit OR healing time)  KL-4: (medicinal plants OR herbal medicine OR plant extract) AND (wound healing) AND (effectiveness OR clinical trial OR efficacy) | 4  3  13  8 |

**Supplementary Table 2:** Risk Assessment of Studies included in investigating the Clinical Effectiveness of Traditional Polyherbal Formulations for Wound Healing

| **Author** | **Domain 1: Bias arising from the randomization process** | **Domain 2: Bias due to deviations from intended interventions** | **Domain 3: Bias due to missing outcome data** | **Domain 4: Bias in measurement of the outcome** | **Domain 5: Bias in selection of reported results** | **Overall Risk of Bias** |
| --- | --- | --- | --- | --- | --- | --- |
| Huang et al. [29] | Low | Low | Low | Low | Low | Low |
| Ko et al. [30] | Some Concerns | Some Concerns | Some concerns | Low | Low | Some Concerns |
| Leung et al. [31] | Low | Some Concerns | Low | Low | Low | Low |
| Li et al. [32] | Low | Some Concerns | Some Concerns | Low | Low | Some Concerns |
| Li et al. [33] | Low | Some Concerns | Some Concerns | Low | Low | Some Concerns |
| Salahi et al. [34] | Low | Low | Low | Low | Low | Low |
| Sanpinit et al. [35] | Some Concerns | High | Low | Some Concerns | Low | High |
| Viswanathan et al. [36] | Low | Some Concerns | Low | Some Concerns | Low | Some Concerns |

**Supplementary Table 3.** Assessment of population characteristics in relation to Clinical Effectiveness of Traditional Polyherbal Formulations for Wound Healing

| Author | **Age (Years)** | **Sex** | **Duration of Diabetes (Yrs)** | **Wound location** | **Duration of wound (Months)** | **Severity** | **Baseline wound size** |
| --- | --- | --- | --- | --- | --- | --- | --- |
| Huang et al. [29] | **ON101**  57.4 ± 10.6  **Control**  56.6 ± 11.3 | 61F; 175M  **ON101**  29F; 93M  **Control**  32F; 82M | 7.2 ± 13.4  ≤ 10yrs =92  >10yrs=144 | Lower Limb | 7.15±13.4 months  <6 months;165  ≥6 months; 71 | Wagner Grade  1=52  Wagner grade 2=184 | 4.8 ± 4.4 cm²  1-5 cm²=165  >5cm = 69 |
| Ko et al. [30] | **NF3**  74.0 ± 12.0  **Placebo**  72.1 ± 12.4 | **NF3**  4F; 4M  **Placebo**  4F; 4M | **NF3**  7.3 ± 6.2  **Placebo**  11.8 ± 10.9 | Lower Limb  **NF3**  Plantar:1 (12.5%), Toes:3 (38.5%), Foot dorsum:1(12.5%), Ankle: 1 (12.5%), other sites: 2 (25.0%).  **Placebo**  Plantar:1 (12.5%), Toes: 2 (25.0%), foot dorsum: 1(12.5%), Ankle: 3 (38.5%), Other sites: 1 (12.5%) | **NF3**  1.87  **Placebo**  3.5 | Mild DFU | 1 to 25 cm²  **NF3**  0.81cm^2^  **Placebo**  0.33 cm^2^ |
| Leung et al. [31] | **HTG**  66.3 ± 12.6  **Placebo**  68.5 ± 11.1 | **HTG**  15F; 25M  **Placebo**  18F; 22M | **HTG**  8.4 ± 7.6  **Placebo**  12.4 ± 8.8 | Lower Limb  **HGT**  Toes: 62%, Foot Dorsum: 22%, Heel: 5%, Sole: 5%, Other sites: 5%  **Placebo**  Toes: 69%, Foot dorsum: 11%, Heel: 3%, Sole: 9%, Other sites: 9% | **HTG**  1.80 ± 1.89  **Placebo**  2.97 ± 5.66 | N/M | **HTG**  28.7 ± 31.3cm²  **Placebo**  26.7 ± 27.3 cm² |
| Li et al. [32] | **CMG**  54.1 ± 14.8  **WMG**  46.2 ± 13.9 | **CMG**  13F: 18M  **WMG**  15F; 16M | **CMG**  8 years  **WMG**  6 years | Lower Limb | **CMG**  8.64 ± 7.80  **WMG**  7.20 ± 4.92 | >2cm | **CMG**  1654 mm²  **WMG**  1452 mm² |
| Li et al. [33] | 38-80 years  **TYO**  60 ± 13  **SWT**  60 ± 11 | 17F; 31M  **TYO**  6F; 18M  **SWT**  11F; 13M | 9.8 ± 5.5  **TYO**  9.5 ± 4.3  **SWT**  10.0 ± 6.9 | Lower Limb  **Plantar**  TYO 15 (62.5%)  SWT 17 (70.8%)  **Non-Plantar**  TYO (37.5%)  SWT (29.2%) | Median  **TYO**  7 months (3-108)  **SWT**  7.5 months (3-60) | Wagner Grade (1-3)  Neuropathic  **TYO**:13(54.2%)  **SWT**:16(66.7%)  Superficial Infection  **TYO**: 18(75%)  **SWT:**16(66.7%)  Deep Infection  **TYO**: 20.8%  **SWT**: 16.7%  Osteomyelitis  **TYO**: 4.2%  **SWT**: 0% | **TYO** (n=21)  3.8 ± 3.72 cm²  **SWT** (n=18)  5.4 ± 4.95 cm² |
| Salahi et al. [34] | 18 and 80 years  **Dermaheal**  57.9 ± 9.88  **Placebo**  55.6 ± 10.44 | **Dermaheal**  6F; 19M  **Placebo**  8F; 17M | **Dermaheal**  11.0 ± 8.18  Placebo  **12.8 ± 7.28** | Lower Limb  **Dermaheal**  Soles: 8 (32%), Heels: 2 (8%), dorsum: 3(12%), Toes: 9 (36%), Leg: 3 (12%)  **Placebo**  Soles: 2 (8%), Heels: 2 (8%) Dorsum: 7 (28%), Toes: 13 (52%), Leg 1: (4%) | **Dermaheal**  2.33 ± 2.93,  **Placebo**  2.00 ± 2.64 | Wagner Grade (1-3)  **Dermaheal** Grade I:  10 (40%)  Grade II:  15 (60%)  **Placebo**  Grade II (44%) | **Dermaheal**  0.9 ± 1.61 cm²  **Placebo**  1.0 ± 1.49 cm² |
| Sanpinit et al. [35] | ≥18 years (20-80years)  **YaSP**  55.04 ± 1.87  **Control**  54.48 ± 1.60 | **YaSP**  F:5 (20%)  M:20 (80%)  **Control**  F: 11 (44%)  M:14 (56%) | **YaSP**  6.04 ± 0.81  **Control**  10.28 ±1.34 | **Lower Limb**  **YaSP**  Plantar: 14 (56%), non-plantar:11 (44%)  **Control**  Plantar: 24 (96%), non-plantar:1 (4%) | **YaSP**  9.99 ± 1.79  **Control**  4.88 ± 0.73 | Wagner Grade (1-2)  **YaSP**  Grade 1:19 (76%)  Grade 2:5 (20%)  **Control**  Grade 1:21 (84%)  Grade 2: 4 (16%) | **YaSP**  2.66 ± 0.72 cm²  **Control**  3.54 ± 0.78 cm² |
| Viswanathan et al. [36] | **Polyherbal**  59.4 ± 8.6  **SSC**  58.7 ± 4.6 | **Polyherbal** F:7  M:12  **SSC**  F: 2  M:17 | **Polyherbal**  13.7 ± 6.5  **SSC**  12.1 ± 4.4 | **Lower Limb**  **Polyherbal**  Plantar: Forefoot (12, 63.2%), Mid foot (5, 26.3%), Hind foot (2, 10.5%)  **SSC**  Plantar: Forefoot (13, 68.4%), Mid foot (4, 21.1%), Hind foot (2, 10.5%). | **Polyherbal**  0.49 ± 0.45  **SSC**  0.46 ± 0.28 | Wagner Grade (1-3)  **Polyherbal**  Grade I:(5, 26.3%)  Grade II:(5, 26.3%)  Grade III:(9, 47.4%)  **SSC**  Grade I: (6, 31.6%),  Grade II: (7, 36.8%)  Grade III: (6, 31.6%) | **Polyherbal**  Length  4.98 ± 1.5 cm width  3.54 ± 1.4 cm  **SSC**  Length  4.3 ± 1.4 cm  Width  3.0 ± 0.98 cm |

*CMG- Chinese Medicine group; WMG- Western Medicine group; SSC- Silver sulphadiazine cream; HTG-Herbal Treatment group; TYO- Tangzu Yuyang Ointment; SWT- Standard Wound Therapy; FAS- Full Analysis set; mITT: Modified Intention-to-treat (mITT); SSC- Silver Sulphadiazine Cream*

**Supplementary Table 4.** Assessment of Clinical Secondary Outcomes in relation to Clinical Effectiveness of Traditional Polyherbal Formulations for Wound Healing

| **Author** | **Amputation-Related Data** | **Granulation Time** | **Adverse effect** |
| --- | --- | --- | --- |
| Huang et al. [29] | **TEAEs**  ON101 (45.9%) 52/122  Comparator (52.6%) 60/114 | N/M | **TEAEs**  ON101 (62.3%) 76  Comparator (67.5%) 77  **Related TEAEs**  ON101 (5.7%) 7  Comparator (4.4%) 5  **Related Serious TEAEs**  ON101 (0%) 0  Comparator (0.9%) 1 |
| Ko et al. [30] | N/M | N/M | NF3 (12.5%) 1  Placebo (37.5%) 3 |
| Leung et al. [31] | Limb Salvage Rate  85% limb rescue rate in HTG compared with placebo group | Granulation maturation time was measured as the duration before end-stage skin grafting. A Kaplan-Meier plot and a log-rank test were used for analysis to compare the duration in each group | N/M |
| Li et al. [32] | **Surgical debridement**  CMG: (0%; 0/27)  WMG: 30.7% (8/26) | N/M | **CMG**  3.7% (1/27) |
| Li et al. [33] | N/M | N/M | **Total Adverse Events**  TYO (29%); SWT (29%)  **Study-Related Adverse Events**  TYO (14%); SWT group (21%)  **Specific Adverse Events**  TYO: Infection (3), Pain (1), UGIT (1)  SWT (21%): Infection (5), Pain (1), UGIT (0)  **Serious Adverse Events**  TYO (14%) 4; SWT (7%) 2  **Death**  TYO: 3%, SWT:1% |
| Salahi et al. [34] | **Dermaheal** (n=25)  1 patient (4%)  Placebo (n=25)  3 patients (12%) | N/M | No Reported Adverse Effects |
| Sanpinit et al. [35] | N/M | N/M | N/M |
| Viswanathan et al. [36] | N/M | N/M | No adverse events reported |


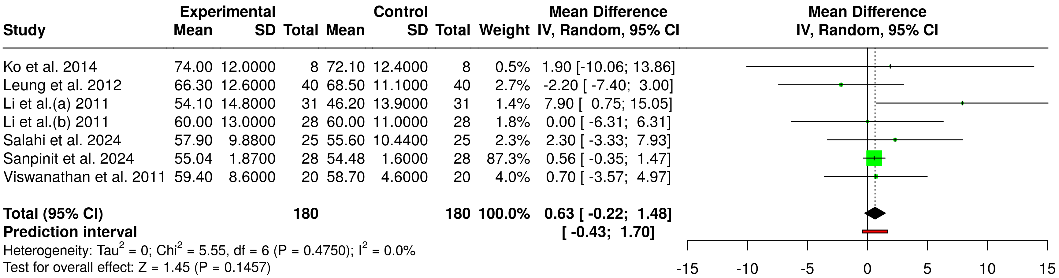

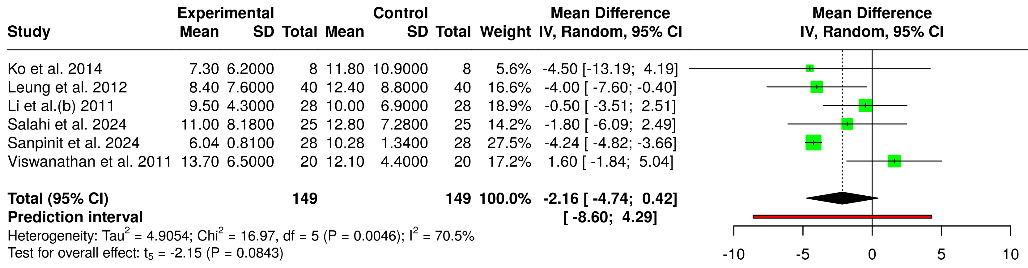


**(B)**

**(A)**


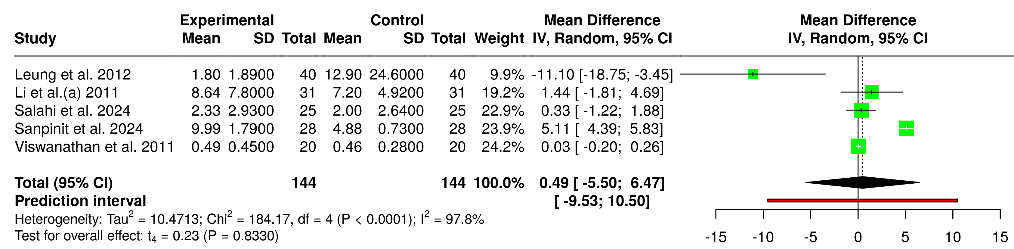

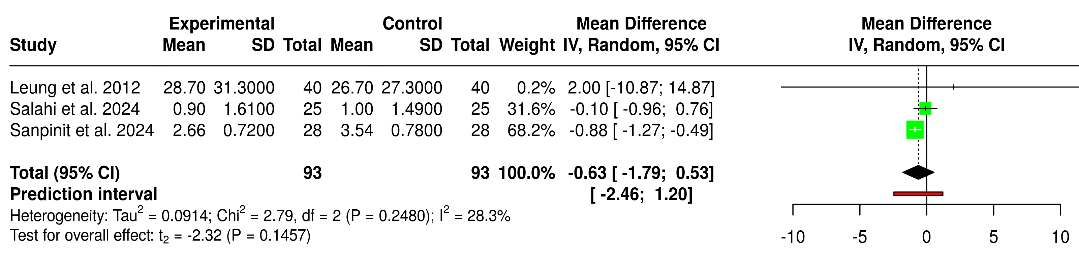


**(C)**

**(D)**


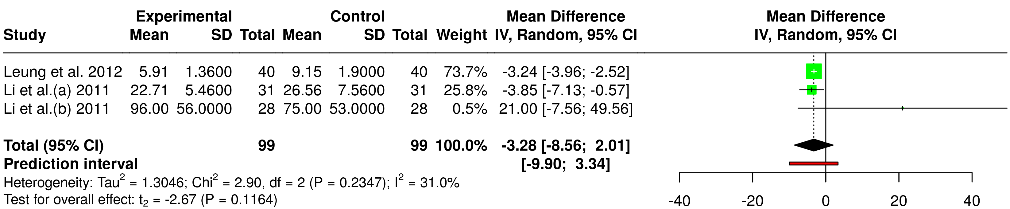

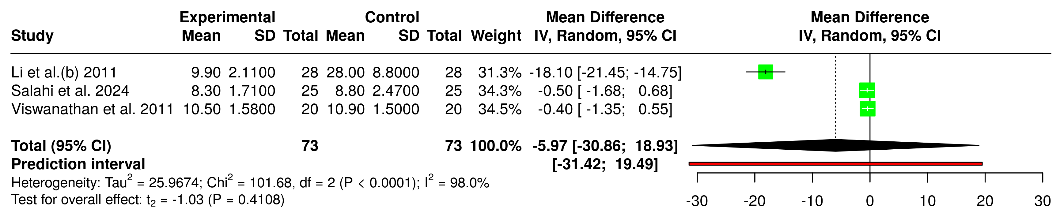


**(F)**

**(E)**

**Supplementary Fig.1.** Forest plot analysis showing the Clinical Effectiveness of Traditional Polyherbal Formulations for Wound Healing compared to the control group on Age (A), Duration of Diabetes (B), Duration of Wound (C), Baseline Wound Size (D), Wound Healing Time (E) and HbA1c Level (F)


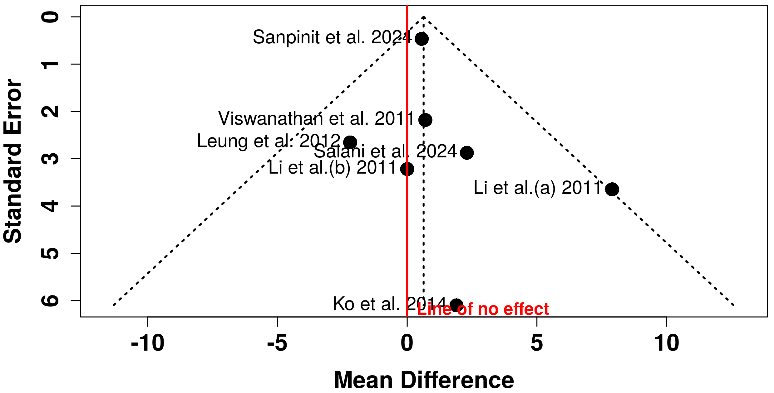

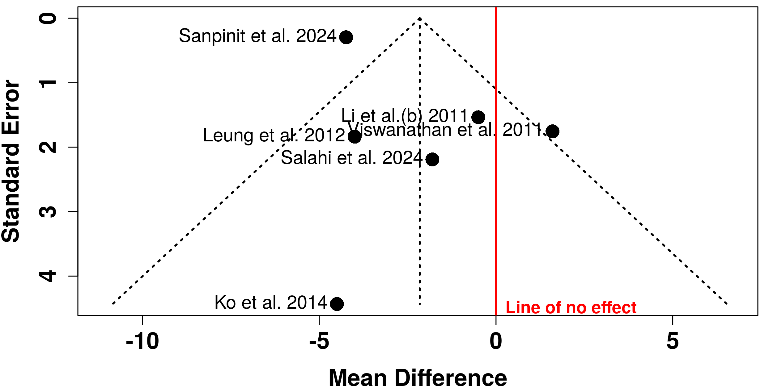


**(B)**

**(A)**


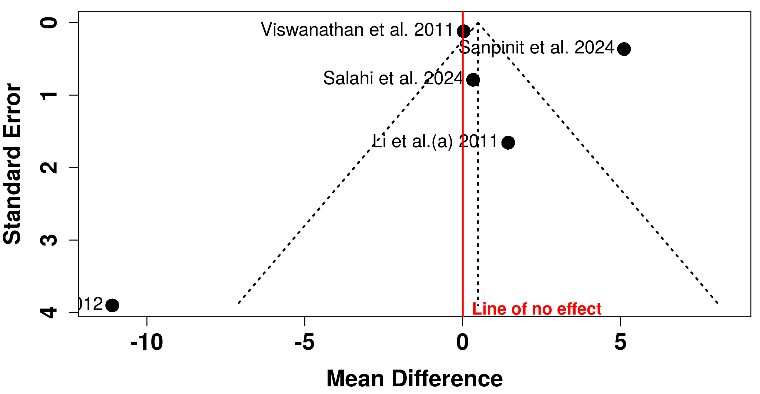

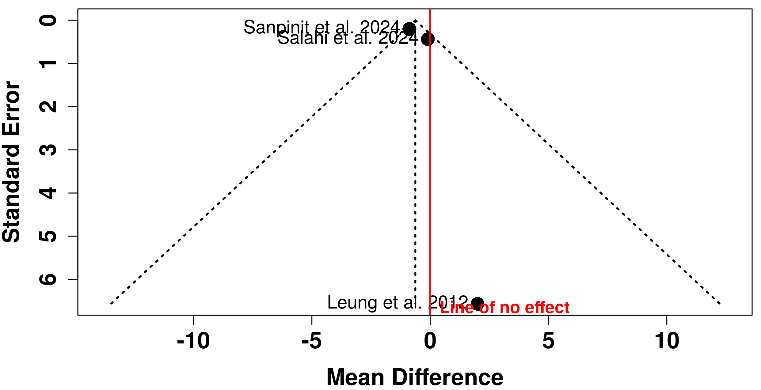


**(C)**

**(D)**


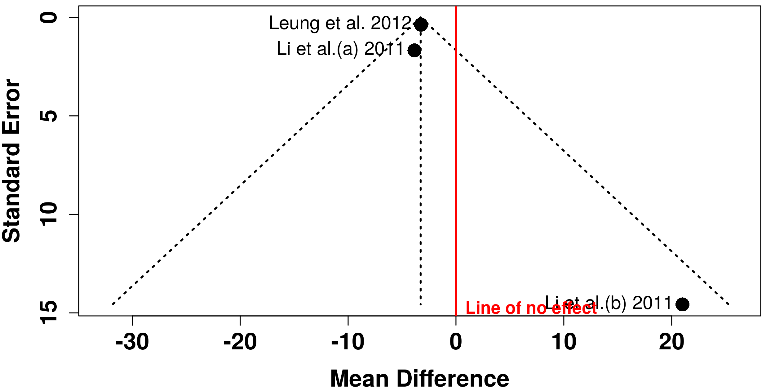

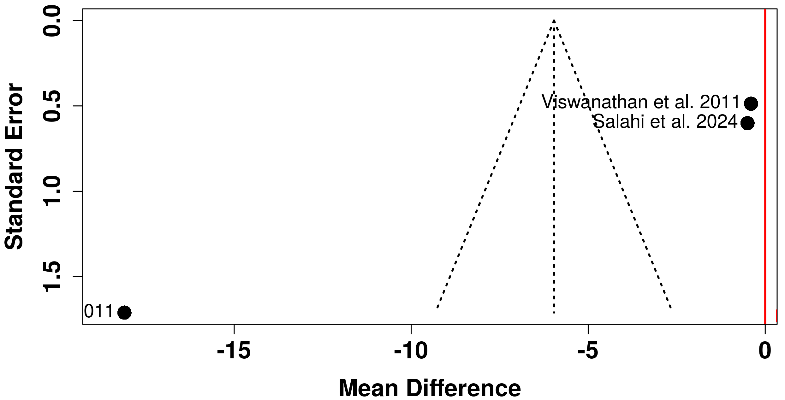


**(E)**

**(F)**

**Supplementary Fig.2.** Funnel plot analysis showing the Clinical Effectiveness of Traditional Polyherbal Formulations for Wound Healing compared to the control group on Age (A), Duration of Diabetes (B), Duration of Wound (C), Baseline Wound Size (D), Wound Healing Time (E) and HbA1c Level (F).

# Supplementary Table 5. GRADE table for Summary of Findings based on meta-analysis of studies on Clinical Effectiveness of Traditional Polyherbal Formulations for Wound Healing

| **Outcome** | **No. of Participants (Studies)** | **Relative/Absolute Effect (95% CI)** | **Certainty of the Evidence (GRADE)** | **Comments** |
| --- | --- | --- | --- | --- |
| Age (years) | 360 (7 RCTs) | MD 0.63 years higher  (–0.39 to 1.66) | ⭑⭑⭑⭑ High | No significant difference in baseline age; low heterogeneity. |
| Duration of diabetes (years) | 298 (6 RCTs) | MD 2.16 years lower  (–4.74 to 0.42) | ⭑⭑⭒ # Low | High heterogeneity (I² = 71%) reduces certainty; baseline variation may affect wound outcomes. |
| Duration of wound (months) | 288 (5 RCTs) | MD 0.49 months higher  (–5.50 to 6.47) | ⭑⭒## Very Low | Very high heterogeneity (I² = 98%) and wide CI; uncertain clinical relevance. |
| Baseline wound size (cm²) | 186 (3 RCTs) | MD 0.63 cm² lower  (–1.79 to 0.53) | ⭑⭑⭑⭒ Moderate | No heterogeneity; results precise but small sample size. |
| Wound healing time (days) | 198 (3 RCTs) | MD 3.28 days shorter  (–8.56 to 2.01) | ⭑⭑⭑⭒ Moderate | Consistent direction of effect but not statistically significant. |
| HbA1c (%) | 146 (3 RCTs) | MD 5.97% lower  (–30.86 to 18.93) | ⭑⭒ ## Very Low | Extremely high heterogeneity (I² = 98%); indirect outcome for wound healing. |

GRADE Working Group Grades of Evidence:

⭑⭑⭑⭑ High certainty: Very confident the true effect lies close to that of the estimate.

⭑⭑⭑⭒ Moderate certainty: True effect is likely close to the estimate, but there is a possibility it is substantially different.

⭑⭑⭒# Low certainty: True effect may be substantially different from the estimate.

⭑⭒# Very low certainty: Very little confidence in the effect estimate
